# Phylogenetic estimation of diversity-dependent biogeographic rates using deep learning

**DOI:** 10.64898/2026.02.17.706216

**Published:** 2026-02-18

**Authors:** Albert C. Soewongsono, Michael J. Landis

**Affiliations:** * Department of Biology, Washington University in St. Louis, Rebstock Hall, St. Louis, Missouri, 63130, USA; † Department of Biology, Washington University in St. Louis, Rebstock Hall, St. Louis, Missouri, 63130, USA

**Keywords:** biogeography, phylogeny, diversification, carrying capacity, competition, deep learning

## Abstract

Ecological theory predicts that local species richness can influence biogeographic rates of speciation, extinction, and dispersal. For instance, increasing the number of competing species within a region may cause local speciation and dispersal rates to decrease but local extinction rates to increase, inducing a carrying capacity for local species richness. In this article, we introduce a fully generative, event-based phylogenetic diversification model, called DDGeoSSE, that allows diversity-dependent effects of local species richness to modulate biogeographic rates of diversification and range evolution. DDGeoSSE can accommodate and test a variety of alternative diversification scenarios that involve positive, negative, and neutral interactions among sympatric species for speciation, extinction, and dispersal. We derive mathematical and statistical properties of biogeographic outcomes generated by this model, such as the carrying capacity for a clade at equilibrium, which we validate through simulation. Because diversity-dependent phylogenetic models typically do not have tractable likelihood functions, we use deep learning with phyddle to perform parameter inference and model selection. Separately applying DDGeoSSE to Caribbean *Anolis* lizards and cloud forest-dwelling *Viburnum* plants, we find evidence that local species richness plays a significant role in shaping diversification dynamics for both clades.

## Introduction

Biologists have long puzzled over two interrelated problems concerning spatial patterns of biodiversity. On one hand, species richness is unevenly distributed in space, with some regions containing far more species than others (Gaston, 2000; Hillebrand, 2004; Mittelbach et al., 2007). On the other hand, species richness at both local and global scales does not increase indefinitely (Van Valkenburgh, 1999; Phillimore and Price, 2008; Pires et al., 2017), where ecological forces, such as competition, are predicted to limit unbounded growth (Macarthur and Wilson, 1967; van Valen, 1973). The exceptionally clear fossil record for marine invertebrates also documents that taxon richness varies among habitats (Bambach, 1977) and latitudes (Jablonski et al., 2006) over time. In addition, periods of higher richness tend to instigate faster extinction and slower origination rates whereas, inversely, faster origination and slower extinction rates accompany periods of lower richness (Alroy, 2008). Learning what dynamics govern the gain, loss, and maintenance of species diversity in space, particularly over long timescales, is therefore a question of central importance to ecologists and evolutionary biologists alike.

To study diversification, researchers have designed a variety of phylogenetic models that describe how clades accumulate species richness with time. Beginning with the pioneering work in the 1990s by Sean Nee and collaborators (Nee et al., 1992, 1994), a myriad of birth-death models in phylogenetics have been developed over the last several decades (Stadler, 2013; Morlon et al., 2024). Building upon the pure birth process of Yule (1925), which assumes a constant speciation (birth) rate and no extinction, researchers have enhanced the realism of diversification models by accounting for time heterogeneity, variation across lineages, species’ intrinsic and extrinsic factors, such as species age (Rabosky and Lovette, 2008; Hagen et al., 2015; Soewongsono et al., 2022), inherited traits (Maddison et al., 2007; FitzJohn, 2010; Beaulieu and O’Meara, 2016), and environmental conditions (Condamine et al., 2013; Mazet et al., 2023), among many others. One such development is the Geographic State Speciation-Extinction (GeoSSE) framework (Goldberg et al., 2011), which simultaneously models how speciation, extinction, and dispersal rates are shaped by the geographical range of each species as it evolves. Since then, researchers have further improved GeoSSE to allow unobserved (hidden) characters (Caetano et al., 2018), regional features (Landis et al., 2022), or both (Quintero et al., 2023), to modulate biogeographic rates and frame how we test biogeographic hypotheses of diversification.

Despite these advances, most existing diversification models allow species richness to increase with time with unbounded growth, contradicting predictions made by the equilibrium theory of island biogeography (Macarthur and Wilson, 1967): that, all else being equal, local increases in species richness should tend to increase an island’s extinction rate while decreasing its speciation and immigration rates, inducing an equilibrium level of species richness when the two rates are equal. However, such models of island biogeography are typically deterministic (no randomness of the timing and placement of biogeographic events), phenomenological (island area is used as a proxy to govern competition, rather than actual numbers of species), and non-phylogenetic (they do not use any explicit representation of shared common ancestry when considering historical dynamics).

Relatively few investigators have designed diversity-dependent phylogenetic models of biogeography for measuring carrying capacities (reviewed in Morlon et al., 2024). Rabosky and Glor (2010) innovated a diversity-dependent extension to the Multistate Speciation-Extinction model (MuSSE; FitzJohn, 2012) that assumes each species occupies one island at any instant, and allows island-specific evolutionary rates to decrease linearly with time (to approximate increasing levels of species richness) using a single carrying-capacity parameter. The Dynamic Assembly of Islands through Speciation, Immigration and Extinction framework (DAISIE; Valente et al., 2015, 2017, 2020; Xie et al., 2023) models the assembly of species between a mainland and an island system, using diversity-dependent speciation and immigration rates that decrease linearly with the actual number of species present in the island system (Valente et al., 2015). DAISIE is ideally suited for mainland-island systems, as it does not model dispersal among islands, diversification within the mainland region, or the general existence of widespread species. DAISIE and the Rabosky-Glor model possess other desirable properties, such as the ability to forbid certain interisland dispersal events (Rabosky and Glor, 2010) or to estimate carrying capacity parameters simultaneously from multiple clades (Valente et al., 2015, 2017). However, both frameworks use a relatively limited set of events to model biogeographic history, and assume all biogeographic processes (speciation, extinction, dispersal) are governed by a single carrying capacity parameter, which together limits their ability to determine whether diversity has the same or different impacts on those three processes.

Building upon previous advances, we designed a new Diversity-Dependent GeoSSE (DDGeoSSE) model, which retains the flexibility of the GeoSSE framework (Goldberg et al., 2011) while allowing local species diversity to influence rates of evolution, either positively or negatively. DDGeoSSE is fully generative, meaning it can simulate phylogenetic trees and the evolution of species ranges under a variety of diversity-dependent scenarios. Unlike existing models, our model explicitly accounts for local species diversity (the actual number of species present) in each region of the system. In addition, rather than assuming that a carrying capacity exists by treating it as a parameter in the model’s rate equations, equilibrium diversity (analogous to carrying capacity) is a product from the data-generating DDGeoSSE process. As such, we derive theory to measure the equilibrium diversity under various diversity-dependent scenarios using rate parameters of the model. To characterize the relationships between phylogenetic and biogeographic patterns with diversity-dependent processes, we explore tree-shape statistics generated under our model, including metrics that capture tree balance, the distribution of branching times, and mean range sizes. These statistics provide a way to compare complex structures such as phylogenetic trees (Fischer et al., 2023). As shown in previous studies, they are useful for analyzing branching patterns and changes in diversification rates (Aldous, 1996; Pybus and Harvey, 2000), classifying diversification models (Aldous, 1996, 2001), and testing model adequacy (Schwery et al., 2023).

We were also interested in applying DDGeoSSE for inference tasks, such as estimating model parameters and performing model selection. However, as models become more complex, it becomes increasingly challenging to derive corresponding likelihood functions that are analytical and tractable (Xie et al., 2023), where DDGeoSSE is no exception. This presents a problem, as likelihood-based estimation (e.g., standard maximum likelihood and Bayesian inference) typically requires an explicit likelihood function under the given model. Fortunately, deep learning has shown promising results as an alternative tool for likelihood-free inference, particularly in phylodynamic applications (Voznica et al., 2022; Lambert et al., 2023; Thompson et al., 2024; Qin et al., 2025). Using simulated datasets, we trained several neural networks to make DDGeoSSE predictions, using a deep learning pipeline implemented in the phylogenetics software phyddle (Landis and Thompson, 2025), for parameter estimation and model selection. We then applied our DDGeoSSE networks to two empirical timetrees: (1) the phylogeny of 158 *Anolis* lizard species occupying Caribbean island regions (Poe et al., 2017), and (2) the phylogeny of 38 species of cloud forest-dwelling *Viburnum* plants in the *Oreinotinus* clade (Donoghue et al., 2022).

## Methods

### Orientation

**Figure 1:**
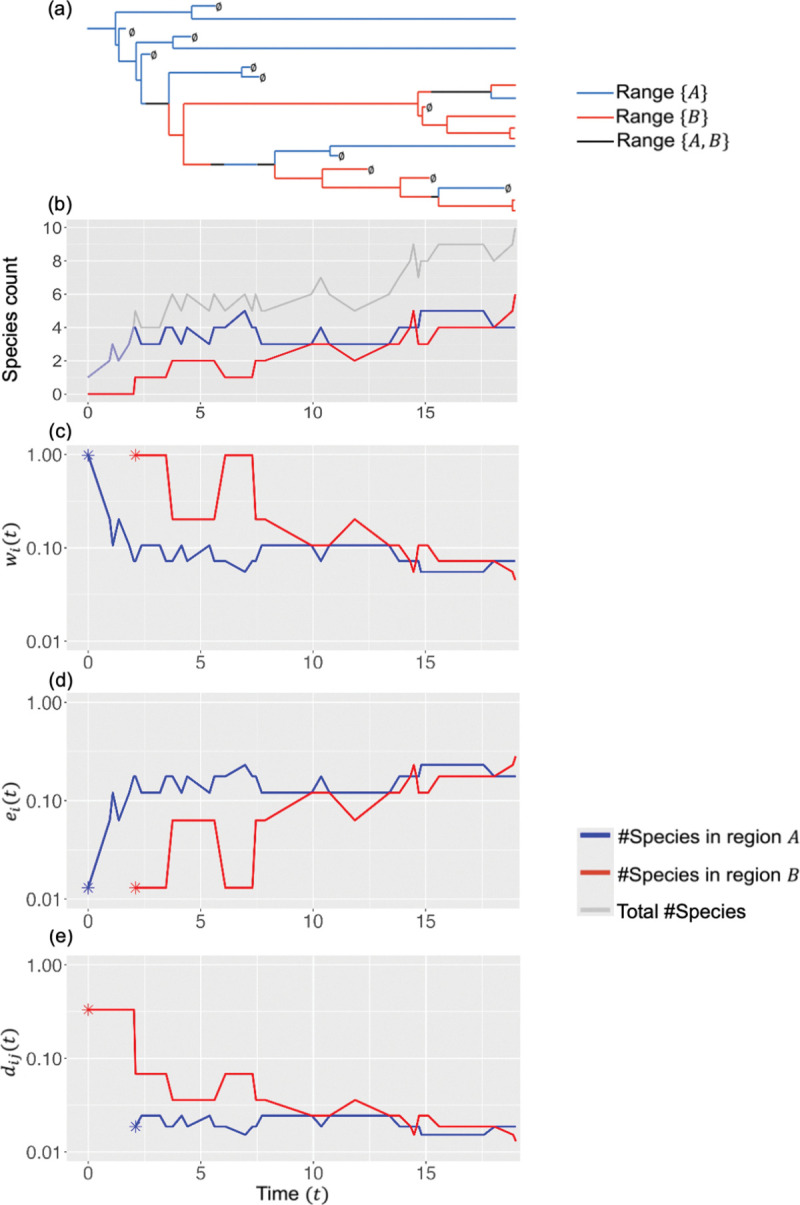
Cartoon of Logarithmic DDGeoSSE model dynamics. (a) Phylogenetic tree with extant taxa that evolved among regions A and B. Blue represents species that only occupy region A, red represents species that only occupy region B, black represents species that occupy both regions, and ∅ represents species extinction. (b) The number of species in region A (blue), in region B (red), and in either region (gray) over time. (c) Within-region speciation rates in region A (blue) and region B (red) over time. (d) Extinction rates in region A (blue) and region B (red) over time. (e) Dispersal rate from region B into A (blue) and from region A into B (red) over time. The blue and red start in panels (c)-(e) indicate the start times when regions A and B (respectively) contain 1+ species, corresponding to non-zero rates (in linear scale) for each process. Species richness in regions A and B both reach local equilibria as time progresses, with increasingly stable biogeographic rates.

We introduce the DDGeoSSE model, which extends the GeoSSE model (Goldberg et al., 2011) to allow local species counts to influence regional rates of speciation, extinction, and dispersal. In turn, this can create distinct phylogenetic and biogeogeographic patterns, such as regional turnover events, as illustrated in Figure 1. For readers unfamiliar with GeoSSE, we provide a description of the model in the Appendix (Supp. Fig. 6). Here, we briefly explain how the DDGeoSSE model behaves, and then outline how the Methods section is structured.

To associate local species counts with the absolute rate for each biogeographic process, the DDGeoSSE model multiplies a base rate parameter, $\rho_{p}$, with a diversity-dependent rate factor, $m_{p}^{D}(i,t)$, in region i for process p∈{d,e,w,b}, corresponding to dispersal, extinction, within-region speciation, and between-region speciation. For example, the extinction rate in region i at time t is written as

$$e_{i}(t)=\rho_{e}\times m_{e}^{D}(i,t).$$

The rate factors for extinction and within-region speciation are defined in terms of single regions, whereas dispersal and between-region speciation are defined in terms of multiple regions. While the base rate parameter, $\rho_{p}$, has a constant value, the rate factor, $m_{p}^{D}(i,t)$, is governed by an estimated diversity-dependent effect parameter, $p^{D}$, that controls the sign and strength for how species diversity in region(s) i at time t influences the rate of the process p (see Table 1). This flexibility allows biologists to test a wide range of hypotheses concerning diversity-dependent biogeography.

After providing the formal probabilistic description of DDGeoSSE, below, we define the equilibrium conditions to attain a desired level of species richness per region, and derive several novel and relevant theoretical results. Next, we explore how different DDGeoSSE modeling scenarios produce predictable changes in tree shape and biogeographic range statistics through simulation experiments. For deep learning-based estimation tasks, we first describe how we simulated training and validation datasets, followed by a detailed description of the pipeline for parameter inference and model selection using phyddle (Landis and Thompson, 2025). Finally, we apply our DDGeoSSE-trained networks to two empirical datasets.

### Logarithmic DDGeoSSE model

Consider a GeoSSE model (Goldberg et al., 2011) defined on a set of n discrete regions in the set of regions, ℛ. If there are multiple species occupying the same region (i.e., in sympatry), DDGeoSSE allows interactions between sympatric species to alter the biogeographic rates any particular species experiences. Moreover, we assume that interactions between two species only occur in the region(s) where they are sympatric. From here on, we refer to our model as *Log-DDG* because the rate function for each process (within-region speciation, between-region speciation, extinction, and dispersal) changes according to the natural logarithm of the total number of species coexisting at a particular time and the diversity-dependent effect parameter. We use a logarithmic function to introduce a diminishing effect for how intensely species diversity influences rates. Biologically speaking, this means diversity-dependence acts on the orders of magnitude instead of on the raw counts of species richness. In the Appendix, we define an alternative formulation using a linear relationship, called *Standard-DDG*, but we did not use it in our analysis, as we found the behavior of this model variant to be exceedingly sensitive to its assigned parameter values.

Next, we define the effect of five diversity-dependent parameters on the event rates for the four canonical GeoSSE event types extinction, within-region speciation, dispersal, and between-region speciation (Table 1). The rate for each species to go extinct in region i at time i, $e_{i}(t)$, is

(1) $$e_{i}(t)=\rho_{e}\times m_{e}^{D}(i,t),$$

where

(2) $$m_{e}^{D}(i,t)={\left({log}_{e}\left(n_{i}(t)\right)+1\right)}^{e^{D}}$$

The extinction rate in region i are unaffected by diversity-dependent effects only if no other species are currently present $\left(n_{i}(t)=1\right)$ or if the effect parameter has no strength $\left(e^{D}=0\right)$.

The absolute within-region speciation rate in region i at time t is

(3) $$w_{i}(t)=\rho_{w}\times m_{w}^{D}(i,t),$$

where

(4) $$m_{w}^{D}(i,t)={\left({log}_{e}\left(n_{i}(t)\right)+1\right)}^{w^{D}}.$$

Similar to extinction, these regional speciation rates experience no diversity-dependent effects if no other species are present $\left(n_{i}(t)=1\right)$ or if the diversity-dependent effect parameter is zero ($w^{D}=0$).

The dispersal rate from region i to j at time i, $d_{ij}(t)$, is defined as

(5) $$d_{ij}(t)=\rho_{d}\times m_{d}^{D}(i,j,t),$$

where

(6) $$m_{d}^{D}(i,j,t)={\left({log}_{e}\left(n_{i}(t)\right)+1\right)}^{d^{D,src}}\times {\left({log}_{e}\left(n_{j}(t)+1\right)+1\right)}^{d^{D,\mathrm{dest}}}.$$

Both $d^{D,src}$ and $d^{D,\mathrm{dest}}$ are the diversity-dependent effect parameters defined on the source region i and the target region j, respectively. Compared with extinction and within-region speciation, there are numerous ways for dispersal to experience no diversity-dependent effects, including cases in which no other species inhabit the source region and/or the destination region has no species, as well as cases in which the source and/or destination effect parameters have values of zero.

The between-region speciation rate, $b_{\ell }^{k}(t)$, gives rise to two new species with ranges k∈𝒮 and ℓ∈𝒮, respectively, from an ancestral species with range m∈𝒮 at time t, where 𝒮 is the set of possible species ranges. We define the absolute rate as follows:

(7) $$b_{\ell }^{k}(t)=\rho_{b}\times f_{b}\left(k,\ell ,t;m_{b}^{D}\right),$$

where

(8) $$f_{b}\left(k,\ell ,t;m_{b}^{D}\right)\propto {\left[\sum_{\left(i,j)\in E(k,\ell \right)}{\left[\frac{m_{b}^{D}(i,\;j,\;t)\;+\;m_{b}^{D}(j,\;i,\;t)}{2}\right]}^{-1}\right]}^{-1}$$

is the range split score function that depends on time t, species ranges k and ℓ, and

(9) $$m_{b}^{D}(i,j,t)={\left({log}_{e}\left(n_{i}(t)n_{j}(t)\right)+1\right)}^{b^{D}}$$

is the diversity-dependent rate factor for species in regions i and j. The range split score function, $f_{b}$, is used to compute the relative rates of between-region speciation for different rate splits (Landis et al., 2022). A higher range split score means that the particular range split pattern is more likely to occur. Meanwhile, E(k,ℓ) represents the minimal set of edges that must be separated to fully isolate all nodes in range k∈𝒮 from all nodes in range ℓ∈𝒮, and the diversity-dependent rate factor $m_{b}^{D}(i,j,t)$ can be thought of as an edge weight that measures the connectivity between region i and j at time t. The edge weight accounts for the numbers of different species currently present in both regions at time t. Note that when $f_{b}\left(k,\ell ,t;m_{b}^{D}\right)=1$, Eq. (8) reduces to the diversity-independent base between-region speciation rate, $b_{\ell }^{k,I}(t)=\rho_{b}$ when its effect parameter is zero $\left(b^{D}=0\right)$ or when the species is alone in all regions it occupies $\left(n_{i}(t)=n_{j}(t)=1,\forall i,j\in E(k,\ell )\right)$.

### Local equilibrium diversity

We give a mathematical definition of local equilibrium diversity in Definition 1, below. Biologically, this equilibrium can be thought of as a stationary phase of the process, in which the mean number of local species remains unchanged over time under a given model of diversification. Importantly, local diversity is tied to each individual region, rather than being the global sum of diversity across all regions. In addition, local equilibrium diversity in DDGeoSSE is not a parameter that directly impacts the rates of the evolutionary processes, but instead it is an emergent property (e.g., expected value) of the data-generating process. This formulation may help reduce bias in statistical inference since we do not target the equilibrium diversity as an estimated parameter (Etienne et al., 2016). We also note that our model does not assume that speciation, extinction, and dispersal are collectively governed by a single, shared diversity-dependence (carrying capacity) parameter; rather, each process may contribute different amounts to the aggregate level of equilibrium diversity within a region.

#### Definition 1.

Given a diversity-dependent GeoSSE model with range state space 𝒮 and region state space ℛ, we define the local equilibrium diversity in region i as

(10) $$n_{i}^{*}=\underset{t\to \infty }{lim}n_{i}(t),$$

where $n_{i}(t)$ is the solution of the following balance equation

(11) $$w_{i}(t)+\sum_{\begin{array}{c} j\in R\\ j\neq i \end{array}}d_{ji}(t)=e_{i}(t).$$

**Figure 2:**
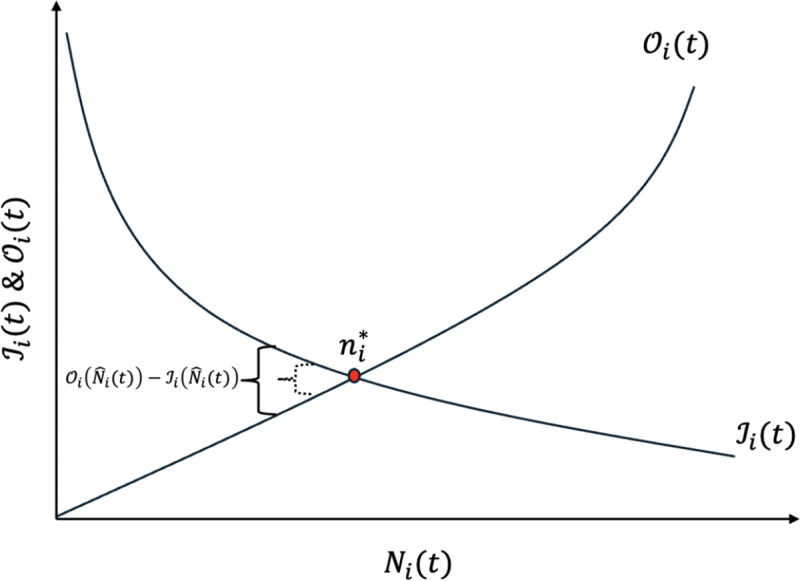
A visualization of the relationship between ${𝒪}_{i}(t)$ and ${ℐ}_{i}(t)$. The equilibrium solution $n_{i}^{*}$ for region i is obtained when ${𝒪}_{i}(t)={ℐ}_{i}(t)$ (as shown by the curly brackets where the difference between the two quantities gets smaller and smaller after each infinitesimal time step Δt until it reaches the intersection point). Note that this figure shares a similar resemblance to the theory of island biogeography from Macarthur and Wilson (1967).

The left-side of Eq. (11) can be thought of as the total incoming rate to region i, ${ℐ}_{i}(t)$, and the right-side of the equation can thought of as the total outgoing rate from region i, ${𝒪}_{i}(t)$. The balance equation can also be viewed as in Figure 2. That is, $n_{i}^{*}$ is such that

(12) $$\frac{dN_{i}(t)}{dt}={𝒪}_{i}\left(N_{i}(t)\right)-{ℐ}_{i}\left(N_{i}(t)\right)=0.$$

Note that the balance equation is general, meaning that it can be used for any diversity-dependent model that accounts for the same evolutionary processes and local species counts (see also Chevin (2016) for a similar formulation of the balance equation in the BiSSE model (Maddison et al., 2007), and Soewongsono and Landis (2024) for a ClaSSE implementation (Goldberg and Igić, 2012)).

#### Lemma 1. (Existence of solution)

Given the balance equation described in Eq. (11) on an |ℛ|–*region system*, where |ℛ| denotes the number of discrete regions, and let $n_{i}(t)$ denote the number of local species diversity at time $t\in R_{\geq 0}$, defined on a closed interval $\left[t_{a},t_{b}\right]\subset R_{\geq 0}$ where $t_{a}<t_{b}$. Assume further that $n_{i}^{*}=n_{j}^{*}$, ∀i,j∈ℛ. Then, there exists $n_{i}^{*}$ that solves the balance equation if and only if $f\left(t_{a}\right)f\left(t_{b}\right)<0$ where

(13) $$f\left(t\right)=w_{i}\left(t\right)+\sum_{\begin{array}{c} j\in R\\ j\neq i \end{array}}d_{ji}\left(t\right)-e_{i}\left(t\right),$$

and $n_{i}^{*}$ denotes the local equilibrium diversity in an arbitrary region i.

The proof of Lemma 1 follows directly from the Bolzano’s theorem using the fact that $w_{i}(t)$, $e_{i}(t)$ and $d_{ji}(t)$ are logarithmic functions, which are continuous on the non-negative real numbers.

Note, also, that we assume all regions each have their own local equilibrium diversities that target the same value, due to how the model is parameterized; otherwise, the balance equation becomes under-determined and no unique solution exists. However, this assumption can be relaxed if biologists have prior knowledge or intuition about the values of rates or $n_{j}^{*}$ for the other regions in the system.

Next, we derive the analytical solution for the local equilibrium diversity, $n_{i}^{*}$, assuming that diversity-dependence acts on all processes relevant to the balance equation described in Definition 1, namely within-region speciation, species dispersal, and species extinction (we refer to this as the *full process*). However, derivations under the full process require some constraints to model parameters to obtain analytical solutions, because the exponent terms (i.e., the diversity-dependent effect parameters) in the balance equation are not always integers, and therefore the equation is not a polynomial. Moreover, considering only polynomial cases, no general closed-form solution exists for polynomials of degree five or higher (Abel–Ruffini theorem). Instead, a numerical solution for $n_{i}^{*}$ can be found under the unconstrained settings for the full process, such that the following is true

(14) $$\left|f\left(t^{*}\right)\right|<\epsilon ,\;\text{for}\;\text{some}\;\epsilon >0,t^{*}\in [a,b],$$

where $f\left(t^{*}\right)$ is defined in Lemma 1 and ϵ is chosen to be arbitrarily close to zero.

#### Lemma 2.

Given the balance equation described in Eq. (11) and |ℛ| denotes the number of discrete regions in the system, the local equilibrium diversity in region i under the full process with no diversity-dependent effect on dispersal in the source region, $\left(d^{D,\mathrm{src}}=0\right)$, is given by

(15) $$n_{i}^{*}=exp\left\{{\left[\frac{\rho_{w}\;+\;\rho_{d}(|ℛ|\;-\;1)}{\rho_{e}}\right]}^{\frac{1}{e^{D}-y}}-1\right\},e^{D}\neq y;\rho_{e}\neq 0,$$

assuming $n_{i}^{*}\gg 0$ and $w^{D}=d^{D,\mathrm{dest}}=y$ under Log-DDG model.

#### Proof:

Proof of Lemma 2 is given in Appendix.

#### Lemma 3.

Given the balance equation described in Eq. (11) and |ℛ| denotes the number of discrete regions in the system, the local equilibrium diversity in region i under the full process with time-varying dispersal rate into region i is given by

(16) $$n_{i}^{*}=exp\left\{{\left[\frac{\rho_{w}\;+\;\rho_{d}\left(\left|ℛ\right|\;-\;1\right){\left(\log\left(n_{j}^{*}\right)\;+\;1\right)}^{x}}{\rho_{e}}\right]}^{\frac{1}{e^{D}-x}}-1\right\},e^{D}\neq x;\rho_{e}\neq 0,$$

assuming $n_{i}^{*}\gg 0$ and $w^{D}=d^{D,\mathrm{dest}}=d^{D,\mathrm{src}}=x$ under the Log-DDG model. Note $n_{j}^{*}=n_{i}^{*}$, ∀j≠i, since the model assumes equal local equilibrium diversity between i and j.

#### Proof:

Proof of Lemma 3 is given in Appendix

**Figure 3:**
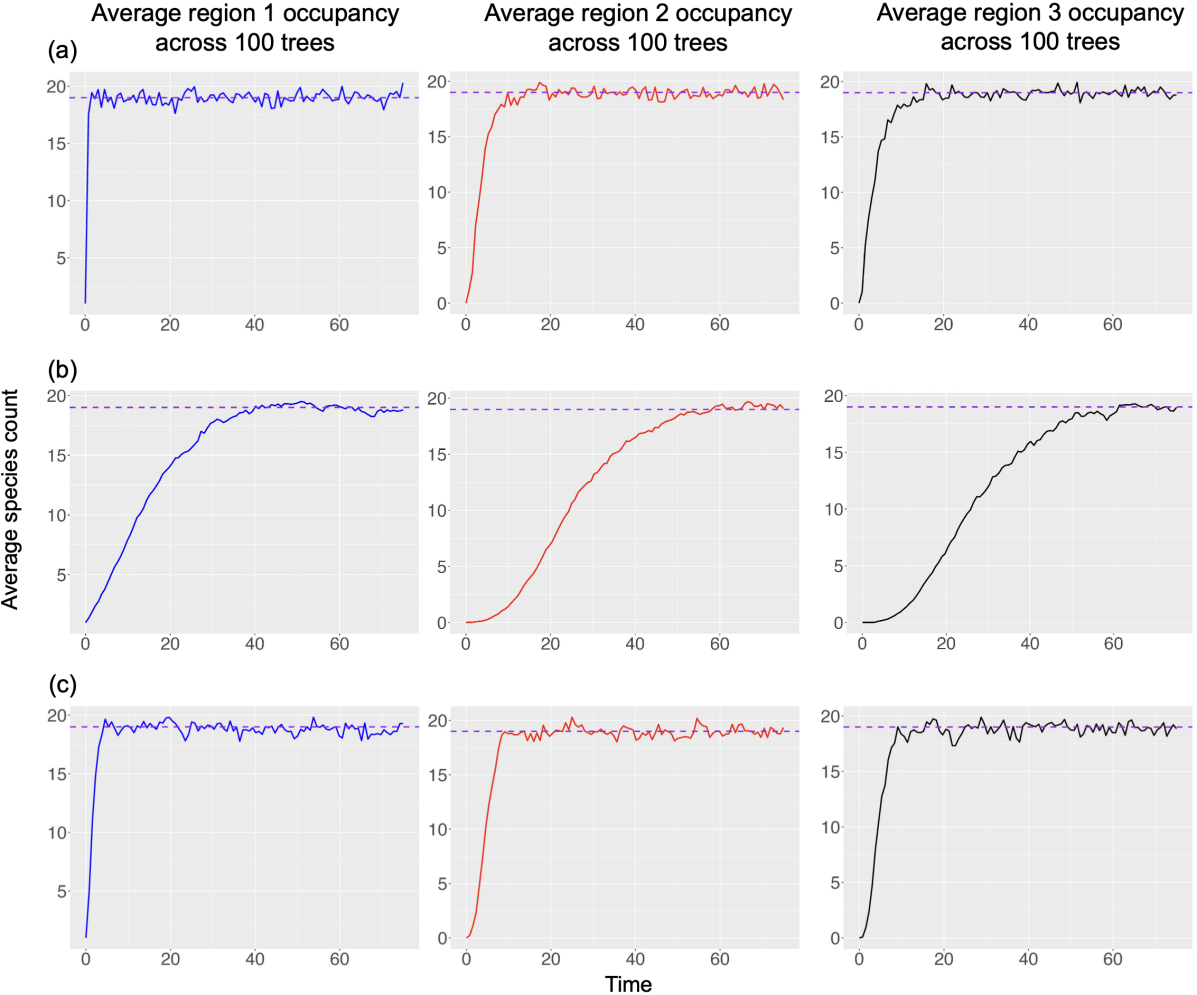
Mean trajectories of $n_{i}(t)$, for each of three regions and averaged across 100 replicates. Trajectories were simulated under (a) a fully unconstrained Log-DDG model according to Lemma 1 with parameter values equal to $\rho_{w}=15$, $\rho_{e}=0.03,\;\rho_{d}=0.05$, $\rho_{b}=1$, $w^{D}=-0.5$, $b^{D}=-1$, $d^{D,\mathrm{dest}}=-2$, $d^{D,\mathrm{src}}=-1$, and $e^{D}=4$, (b) a constrained Log-DDG model according to Lemma 2 with parameter values equal to $\rho_{w}=0.610047$, $\rho_{e}=0.01$, $\rho_{d}=0.01$, $\rho_{b}=1$, $w^{D}=d^{D,\mathrm{dest}}=-1$, $d^{D,src}=0,\;b^{D}=0$, and $e^{D}=2$, and (c) a constrained Log-DDG model according to Lemma 3 with parameter values equal to $\rho_{w}=3.730315$, $\rho_{e}=0.03$, $\rho_{d}=0.04$, $\rho_{b}=1,\;w^{D}=d^{D,\mathrm{dest}}=d^{D,\mathrm{src}}=-0.5$, $b^{D}=0$, and $e^{D}=3$. In all scenarios, each region is expected to have local equilibrium diversity, $n_{i}^{*}$, of around 19 extant species. $n_{i}^{*}$ is solved numerically in the fully unconstrained model using Eq. (14). For each tree simulation, we initialize a tree with root species only in region 1.

We demonstrate Lemma 1 by solving for $n_{i}^{*}$ numerically for a given set of parameter values using Eq. (14). Then, we validate this by simulating trees under the same set of parameters to show that $n_{i}^{*}$ matches (Fig. 3(a)). Finally, we demonstrate both Lemma 2 and Lemma 3 by analytically finding sets of parameters that correspond to the same $n_{i}^{*}$ and validate these results through simulation (Figs. 3(b)–(c), respectively).

### Tree statistics for trees simulated under the Log-DDG model

In this section, we explore several tree statistics under the Log-DDG model with diversity-dependence on within-region speciation, extinction, and incoming dispersal (i.e., $w^{D}$, $e^{D}$ and $d^{D,\mathrm{dest}}\neq 0$). For the simulation experiment, we consider our model with 3 regions. We use simulated extant-only trees to compute two tree statistics, including Aldous’ β statistic (Aldous, 1996) for tree topology and the γ statistic (Pybus and Harvey, 2000) for branch lengths. We also examine variation in mean range size and mean number of extant taxa across the simulations.

As for the simulation experiments, we construct a 3-dimensional mesh grid of $w^{D}$, $e^{D}$ and $d^{D,\mathrm{dest}}$. The values for both $w^{D}$ and $d^{D,\mathrm{dest}}$ parameters are defined on [−2, 0] interval. This range of values covers both diversity-independent scenario $\left(w^{D},d^{D,\mathrm{dest}}=0\right)$ and a scenario where rate is decreasing as the number of species occupying a region is increasing ($w^{D},d^{D,\mathrm{dest}}<0$) for both events. Meanwhile, the values for $e^{D}$ are defined on [0, 2] interval, which covers both diversity-independent scenario ($e^{D}=0$) and a scenario where rate is increasing as the number of species occupying a region is increasing ($e^{D}>0$). For each combination of these parameter values, we sample without replacement 50 extant-only trees, conditioned on tree height equals to 100 time unit, using the Log-DDG model. We use the following base event rates across all experiments: $\rho_{w}=\rho_{d}=0.05$ and $\rho_{e}=\rho_{b}=0.02$. We also ignore the case where $w^{D}=e^{D}=d^{D,\mathrm{dest}}=0$ and exclude invalid trees (trees with only 1 or 2 extant taxa). In total, we simulate 49, 950 extant-only trees across all combinations.

To assess the effect of trees generated under weak diversity-dependent effect on tree statistics more rigorously, we perform another experiment where the values of both $w^{D}$ and $d^{D,\mathrm{dest}}$ are drawn from [−0.1, 0] interval, and [0, 0.1] for $e^{D}$. In total, we simulate 6, 200 extant-only trees across all combinations.

### Simulated training and validation datasets

To evaluate how reliably deep learning can infer parameters for a biogeographic model with diversity-dependent effects, we simulate training datasets under various diversity-dependent scenarios of the Log-DDG model and train a separate neural network for each scenario. In all cases, we simulate using five regions and impose diversity-independence on outgoing dispersal and between-region speciation (i.e., $d^{D,src}=b^{D}=0$).

We simulate 50, 000 valid training examples under each submodel defined in Table 2. For each replicate, we record the extant-only phylogenetic tree (in .tre extension), tip states (in .csv extension), and parameter values (in .csv extension). The phylogenetic trees are recorded in the Newick string format, while the tip state files record information about species range at tips following a one-hot encoding, by recording species presence (1) or absence (0) in each region in our 5-region system. The parameter files record the true parameter values from the model for that particular replicate and its model type as described in Table 2.

We perform rejection sampling if we have tree extinction or if the tree does not meet one of the following simulation conditions: (1) minimum number of extant taxa (fixed to 10), (2) maximum number of extant taxa (random variable drawn from a uniform distribution on [100, 300] interval), or (3) simulation time (random variable drawn from a uniform distribution on [70, 100]). These conditions will give trees with random height and number of extant taxa.

The Log-DDG model we analyze assumes each region targets the same level of equilibrium diversity ($n_{i}^{*}$), and no other effect alters event rates among regions besides species diversity. For simulation under the null model (submodel 0), we assume equal rates between regions/ranges for each event process. In each submodel, we draw the base rates ($\rho_{w},\rho_{e},\rho_{d},\rho_{b}$) from a mixture of uniform and loguniform distributions, each from the interval of [10^−4^,1]. Specifically, we simulate 25,000 trees with base rates that are drawn from the uniform distribution, and the other 25,000 trees with base rates that are drawn from the loguniform distribution. Through experiments, we found that this ensures an equal representation of values that span different orders of magnitude. We draw the values of the diversity-dependent effect parameters ($w^{D},e^{D},d^{D,\mathrm{dest}}$) from a uniform distribution on [10^−4^,2] for $e^{D}$, and on [−2,−10^−4^] for $w^{D}$ and $d^{D,\mathrm{dest}}$. This particular choice of ranges ensures us a scenario in which overall extinction rate increases with increasing local species diversity, and both overall within-region speciation rate and species dispersal decrease with increasing local species diversity. We summarize the details on simulation setting for each submodel on Table 3.

### Deep learning method for parameter inference and model selection

We use phyddle (Landis and Thompson, 2025), a pipeline-based software for performing phylogenetic modeling tasks on trees using likelihood-free deep learning approaches, for our analyses. The parameter estimation pipeline consists of five main stages:

- **Simulate (S)**. In this stage, we simulate 50,000 training examples under each submodel described in Table 2, where each tree in our training set has between 10 to 300 extant tips. This range is chosen for two main reasons: (1) larger trees provide greater statistical power for inference, and (2) the number of extant tips on the empirical trees later used in this study fall within this range of simulated tree sizes. Moreover, each simulated tree has an origin age that is between [70, 100] units of time. These bounds are slightly greater than the root ages of the empirical trees used in this study, as empirical trees lack an ‘origin’ species (subtending the root node), whereas our simulations start with a single ancestral species. We use our custom script implemented in Julia (Bezanson et al., 2017) to simulate these datasets. We validate our simulator by comparing tree statistics from trees simulated under a GeoSSE model in Diversitree (FitzJohn, 2012) and our simulator (see the Appendix).
- **Format (F)**. In this stage, we encode our raw simulated data (extant-only trees, tip states, and model parameters) into three types of tensors, namely a phylogenetic data tensor, label tensor, and auxiliary data tensor. The software encodes the phylogenetic data tensor using compact diversity vector (CDV) (Lambert et al., 2023) with associated biogeography states at tips (+S) (Thompson et al., 2024).
- **Training (T)**. We used the standard phyddle neural network architecture for our prediction tasks on parameter estimation. For each dataset simulated under each submodel, we take 5% of the total sample size as our test dataset, and another 5% of the remaining sample size as our validation dataset, and train our network separately for each model. For each training, we train our network using 200 training intervals (epochs) with 2,048 samples in each training batch. We repeat the training procedure using the same dataset for each submodel with the weight decay option enabled (weight_decay = 0.001). In phyddle, this helps improve consistency when making same predictions between networks with similar loss scores. In total, we have 16 different trained networks across eight submodels, described in Table 2, for parameter estimation.
- **Estimate (E)**. In this stage, using our trained network from each submodel, we make predictions on parameters (base rates and diversity effect parameters) for the test dataset, which was excluded from training. Later, we also use the same trained network to make predictions for the empirical datasets.
- **Plot (P)**. Lastly, we visualize the output from phyddle analyses. In this stage, we assess the performance of our trained network and the accuracy of our predictions.

Next, we make predictions on empirical parameter values using the trained network from each submodel described in Table 2 by running the F and E stages on the empirical datasets, and plot the results.

We also used phyddle to predict the best-fitting model among the eight different submodels described in Table 2. We first combined all datasets simulated under each submodel to obtain a dataset of size 400,000. Through experiments, we found that using one-hot encoding instead of integer encoding for model types improved network performance (results not shown). That is, instead of using one variable with integers from 0 to 7 to represent the 2^3^ distinct model-type combinations, we used three binary variables to encode presence-absence of each diveristy-dependent effect. Then, we converted this combined dataset to tensor data by running the F step in phyddle. We then separately trained three networks by running the T step on the same dataset to predict whether a particular diversity-dependent feature $-w^{D}$, $e^{D}$, or $d^{D,\mathrm{dest}}$ – was non-zero (‘on’). We then trained 7 additional sets of networks for each of the three parameters to test the correctness of the first set of networks, for 24 (= 3 × 8) networks in total. Given the complexity of the model, we found that using this method (i.e., training for a single categorical prediction and using multiple independent networks) gives a more accurate and consistent prediction results. As such, our model selection predictions used the mean prediction scores across the eight networks for each parameter. Lastly, by running the P step, we obtained a figure illustrating the best fitting model for each empirical data.

Our phyddle configuration files and simulator scripts are provided with the Supplementary Information. The Supplement also displays the phyddle network architecture used for the parameter estimation (Supp. Fig. 26) and model selection analyses (Supp. Fig. 27).

### Empirical data

We use our DDGeoSSE-trained neural networks to analyze Caribbean *Anolis* lizards and cloud forest-dwelling *Viburnum* plants, allowing us to infer the extent to which either clade was shaped by diversity-dependent rates of dispersal, speciation, and/or extinction.

*Anolis* is a adaptive radiation of roughly 380 neotropical lizard species, with more than half of its species inhabiting the Caribbean islands. Convergent evolution has repeatedly produced similar communities of anole ecomorphotypes within and among different islands (Losos et al., 1998), where ecological interactions among anole species may be shaping regional patterns of species richness (Losos and Schluter, 2000). Later work by Rabosky and Glor (2010), using their modified MuSSE model, found that biogeographical speciation rates in each island slowed over time, attributing the cause to diversity-dependent deceleration. To test whether regional diversity influences *Anolis* biogeography, we reduced and re-analyzed the dataset of Poe et al. (2017) to obtain a dataset of 158 island species with ranges associated with five Caribbean regions: the Lesser Antilles, Puerto Rico, Cuba and the Caymans, Hispaniola, and Jamaica. The crown age of the phylogeny representing this taxon-subset is ≈ 64.9 Ma. Supplementary Figure 20 displays the phylogeny and biogeography we used.

*Oreinotinus*, the second group, is a clade of approximately 40 neotropical plant species, nested within the *Viburnum* radiation (Landis et al., 2021). Earlier work by Donoghue et al. (2022) showed that *Oreinotinus* likely dispersed along a series of montane cloud forests, from North America into South America, repeatedly evolving the same set of leaf ecomorphotypes in each region. Their work found almost no evidence for back-dispersal into previously colonized regions, suggesting inbound dispersal rates might increase with regional diversity. To test this hypothesis, we re-analyzed the *Oreinotonus* time tree and biogeographical ranges published by Donoghue et al. (2022), which contained 41 species spanning 12 regions, but modified it so that it was compatible with our neural networks trained for 5-region systems. Our modification removed an outgroup species (*V. dentatum*) in Eastern North America and two Caribbean species (*V. alpinum* and *V. villosum*), leaving 38 species in our tree. We also combined the 10 remaining regions into 5 regions, using adjacency and shared history as criteria for merging: (1) merging Eastern, Central, and Western Mexico, (2) merging Oaxaca, Chiapas, and Central America, (3) keeping Costa Rica and Panama together, (4) merging Western, Central, and Eastern Colombia and Venezuela, (5) and merging Southern Colombia, Northern and Southern Ecuador, Northern and Southern Peru, Bolivia, and Northern Argentina. The original tree is relatively young (≈ 10.7 million years old) and does not fit with tree ages from the training dataset, so we re-scaled the branch lengths of the tree to be 7 times longer before the analysis, and reverted to the original scale after the analysis. Supplementary Figure 21 displays the phylogeny and biogeography we used.

### Hardware used

All phyddle analyses were performed on a Dell Precision 7920 server with 2× Intel CPUs (112 cores total), 2× Nvidia RTX A4000 GPUs, and 128 GB of system RAM.

## Results

### Tree and range statistics under Log-DDG

**Figure 4:**
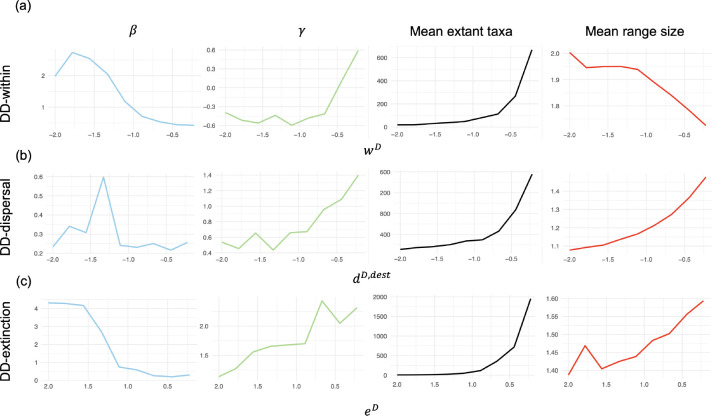
Plots showing changes in the values of various tree statistics from simulated trees drawn from model with diversity-dependent effect on (a) within-region speciation (“DD-within”), (b) dispersal (“DD-dispersal”), and (c) extinction (“DD-extinction”). The *x*–axis represents the values of each diversity-dependent rate scalar respectively ${(w}^{D},d^{D,\mathrm{dest}},e^{D})$ and the *y*–axis represent the values of the tree statistics.

Increasingly strong effects of diversity-dependence on within-region speciation ($w^{D}<0$), dispersal ($d^{D,\mathrm{dest}}<0$), and extinction ($e^{D}>0$) generate increasingly pronounced phylogenetic and biogeographic patterns in simulated data (Figure 4). Overall, diversity-dependent effects increase tree imbalance (β) and decrease the mean numbers of extant taxa, regardless of which process was considered. However, diversity-dependence produces different trends in mean range sizes and node depths (γ-statistic values) for the three processes. We review such relationships between trends and processes in more detail, below.

For within-region speciation, the β statistic used to measure the degree of tree imbalance relative to Yule trees (β=0) tended to increase (more balance) as diversity-dependence increased (Fig. 4(a), first column). An opposite trend is observed for the γ statistic, which led towards negative γ values (divergence times cluster near the root) as diversity-dependence grew in strength (Fig. 4(a), second column). Increasing diversity-dependence also led to decreased mean numbers of taxa (Fig. 4(a), third column), but larger mean range sizes (Fig. 4(a), fourth column).

Dispersal differed from within-region speciation and extinction in that its β values were apparently unaffected by an increasing negative effect of diversity dependence on incoming dispersal rates (Fig. 4(a), first column). On the other hand, γ values increased as the diversity-dependence effect on incoming dispersal weakened (Fig. 4(b), second column). Mean numbers of extant taxa (Fig. 4(b), third column) and mean range sizes (Fig. 4(b), fourth column) both fell as the effects from diversity-dependence on dispersal grew.

Diversity-dependent extinction produced larger β values (Fig. 4(c), first column), indicating greater tree imbalance, and larger γ values (Fig. 4(c), second column), corresponding to increased turnover, with divergence times clustered near the present. In addition, the mean number of extant taxa (Fig. 4(c), third column) and mean range sizes (Fig. 4(c), fourth column) both decreased with stronger positive diversity-dependent effects on extinction.

### Parameter estimation and model selection for Log-DDG

**Figure 5:**
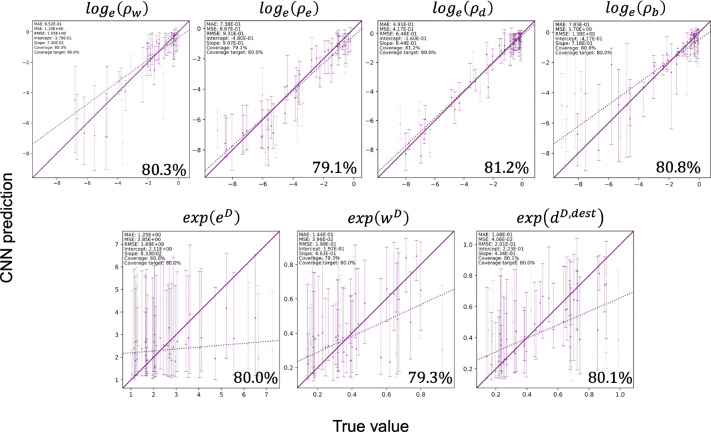
Parameter estimation accuracy against the test dataset simulated under submodel 7. The *x*–axis shows the true parameter values, and the *y*–axis shows the estimated values from CNN. All test data were used for the regression. Of these, point estimates (markers) and 80% CPIs (bars) are shown for 50 examples. Estimates are colored if their CPI contains the true value, and left if not.

We measure the performance of networks trained on datasets simulated under each scenario of the Log-DDG model and the GeoSSE model described in Table 2. For each parameter in a model, we show its conformalized prediction interval (CPI) plot (Romano et al., 2019) from the test dataset with a target coverage level of 80%, meaning that, on average, new predictions will contain the true parameter value 80% of the time, assuming the test and new data are generated by the same model. Simulation results indicate accurate parameter estimation under the most complex diversity-dependent model, where speciation, extinction, and incoming dispersal are all diversity dependent. This is shown by all parameters passing the target coverage level (Fig. 5). We also observe accurate parameter estimation for the other models described in Table 2 (Figs. 11–17 in the Appendix).

While the estimates for all base rate parameters ($\rho_{w},\rho_{e},\rho_{d},\rho_{b}$) are precise (as indicated by tighter CPI intervals), the estimates for the three diversity-dependent effects ($e^{D}$, $w^{D}$, and $d^{D,dest}$) are less certain, as indicated by wider CPI intervals. In particular, the $e^{D}$ estimates are the least precise, as extinction-associated parameters are often the most difficult to estimate accurately (Goldberg et al., 2011; Swiston and Landis, 2024). This is consistent throughout the other models with diversity-dependent extinction (Figs. 12, 15, and 16 in the Appendix). We caution readers when interpreting the estimate of this extinction parameter from data.

Next, using eight independently trained networks on the combined dataset (400,000 trees) from the 8 submodels described in Table 2 for detecting diversity-dependence on each process (within-region speciation, extinction, and dispersal), both networks show accuracies of approximately 73% – 81% for correctly inferring models without diversity-dependence in within-region speciation, and 81% – 87% for correctly inferring models with diversity-dependence in the same process on test dataset. For extinction, both networks achieve accuracies of 76% – 83% for models without diversity-dependence, and 78% – 86% for models with diversity-dependence. Lastly, for dispersal, both networks achieve accuracies of 74% – 80% for models without diversity-dependence, and 83% – 87% for models with diversity-dependence (Figs. 18–19, Appendix).

### Model selection: Anolis tree

We perform model selection on the empirical *Anolis* tree against eight different models defined in Table 2. We find that the dataset is best supported by submodel 7, which has diversity-dependent effects on within-region speciation, extinction, and incoming dispersal, with a slightly lower support for dispersal. The result from model selection is consistent when using the other independently trained networks on the same dataset (Table 5). These results support the hypothesis where higher local species diversity reduces within-region speciation and immigration rates $(w^{D},d^{D,\mathrm{dest}}<0$), and increases the local extinction rate ($e^{D}>0$).

### Model selection: Viburnum tree

We perform model selection on the empirical *Viburnum* tree (*Oreinotinus* clade) against eight different models defined in Table 2. We found that the dataset is supported by submodel 6 that has diversity-dependent effects on within-region speciation and incoming dispersal. However, the support for diversity-dependent dispersal is weaker than that for within-region speciation. The result from model selection is consistent using the other trained network (Table 5). These findings support the hypothesis where higher local species richness reduces both within-region speciation and dispersal rates ${(w}^{D},d^{D,\mathrm{dest}}<0)$, but has no effect on extinction.

We note that one of the auxiliary data tensors (i.e., treeness) shows the *Viburnum* tree as an outlier relative to the training data (Fig. 23 in the Appendix). In this case, the empirical treeness is slightly outside the range of the simulated data (0.43 vs. [0.64, 0.90]). The treeness statistic measures the proportion of internal branch lengths of the tree relative to the overall tree height. The network supports decreased within-region speciation rates as local species diversity increases, which aligns with the *Viburnum* tree having a smaller (more starlike) treeness value.

## Discussion

In this article, we introduce an event-based, fully generative phylogenetic diversification model, called DDGeoSSE, that allows local species diversity to influence biogeographic rates of change. DDGeoSSE is intended to be flexible, and can accommodate a range of evolutionary scenarios in which local species richness influences diversification dynamics through different biogeographic processes. These include scenarios where high regional species diversity can have positive, negative, or neutral feedback on shifts in evolutionary rates for extinction, within-region speciation, between-region speciation, inbound dispersal, and/or outbound dispersal. This framework enables users to simulate species phylogenies and test hypotheses under varying diversity-dependent scenarios.

Our model differs from the few, other existing birth-death models of biogeography that account species richness, such as the fully diversity-dependent DAISIE framework (Valente et al., 2015) or the modified MuSSE model of Rabosky and Glor (2010). Unlike DAISIE, DDGeoSSE models diversity-dependent speciation, extinction, and dispersal rate variation among all regions, and along all branches of the phylogeny, without the need to specify an untracked mainland region. Moreover, unlike the MuSSE model extension, because DDGeoSSE is a generalization of the GeoSSE model, it also allows for cladogenesis in which only one daughter inherits the parent’s range (budding speciation) or both daughters inherit split portions of the parent’s range (allopatric speciation). This, in turn, adds more realism to the model. To the best of our knowledge, DDGeoSSE is the first birth-death model to account for species diversity, historical biogeography, and state-dependent diversification rates with this level of generality (Morlon et al., 2024).

In this work, we also define our concept of local equilibrium diversity for each region and allow users to compute this quantity either numerically in a fully unconstrained model or analytically under some assumptions on the model parameters. This parameter is different from how it is used in other existing models, where it is assumed to be a global parameter across all regions in the system. We can estimate the equilibrium diversity level in a region $\left(n_{i}^{*}\right)$ using our balance equation that models the total rate inflow and outflow locally per region. This balance equation is general, and can be applied to any diversity-dependent model that accounts for actual local species count and same evolutionary processes. While in this study we assume all regions target the same equilibrium diversity level, DDGeoSSE can be configured to allow each region to target different equilibrium levels based on the prior knowledge or predictions of biologists (e.g., due to size or resource variation among regions).

Using simulation experiments, under a scenario with diversity-dependence only impacting within-region speciation, we observe that more species tend to be widespread (Fig. 4(a), fourth column) as the effect strength increases. This is because fewer within-region speciation events occur under this scenario, particularly near towards the present, resulting in fewer young, endemic species. Conversely, as the diversity-dependent effect on within-region speciation weakens, we observe greater numbers of extant species (Fig. 4(a), third column) as our model approaches the standard GeoSSE model (Goldberg et al., 2011), which allows the number of extant species to increase exponentially. In addition, weaker diversity-dependent effects on within-region speciation produces trees with shorter branch lengths towards the present, as measured by the γ statistic (Fig. 4(a), second column). Simulating trees under a scenario with only the positive diversity-dependent effect on extinction, we observe that trees tend to have fewer widespread species, as the diversity-dependent effect increases ((Fig. 4(c), fourth column)). The average number of extant species on trees also decreases, as there are more lineage-level extinction events on trees (Fig. 4(c), third column). Moreover, branch lengths also tend to be shorter near the present when the diversity-dependent effect on extinction is strongest, promoting species turnover (Fig. 4(c), second column). When the diversity-dependent effect acts on the incoming dispersal rate, we see no clear pattern in the tree topology (Fig. 4(b), first column), which is reasonable since dispersal does not directly influence branching patterns. The mean range sizes and number of extant taxa generally increase when the diversity-dependent effect on the incoming dispersal is weak (Fig. 4(b), third and fourth columns). This is because we have more dispersal events, which increases species range size and, indirectly, expands opportunities within- and between-region speciation events, leading to an increase in mean number of extant taxa. In summary, the model can produce characteristic patterns for mean range sizes and the β statistic values, depending on which particular diversity-dependent effect is considered.

Across these different experiments, we also found that reconstructed trees produced under our diversity-dependent model tend to have balanced topologies, as indicated by their β statistics (Fig. 4(a)–(c), first column). This pattern of tree balance is also observed in other diversification models, such as age-dependent models where the speciation rate increases with species age (Hagen et al., 2015; Soewongsono et al., 2022). In contrast with the diversity-dependent model, we do not observe an obvious pattern in tree shape when simulating under very weak to non-diversity-dependent model (Figs. 8 in the Appendix). Furthermore, the distinct pattern on the tree shape observed under our model could be beneficial for model adequacy testing (see Schwery et al. (2023)), validating simulators that implement the same model, and early diagnostics for phylogenetic deep-learning studies (see Landis and Thompson (2025)).

Our model uses a phylogenetic deep-learning approach implemented in phyddle (Landis and Thompson, 2025) to perform inference, such as for parameter estimation and model selection. The accuracy of different parameter estimates varies among for seven different submodels with different combinations of diversity-dependent effects on within-region speciation, extinction, and incoming dispersal, including a model without diversity-dependence in all processes. In general, base rate parameters $(\rho_{d},\;\left.\rho_{e},\rho_{w},\rho_{b}\right)$ are estimated more accurately than the diversity-dependent effect parameters ($e^{D},w^{D},d^{D,\mathrm{dest}}$), with the diversity-dependent extinction effect parameter ($e^{D}$) being the least accurate of all. Independently trained model selection networks, on the other hand, all perform quite well and consistent at correctly classifying which datasets did or did not evolve under different diversity-dependence effects, including extinction (Table 5). Thus, our networks appear to be reliable for distinguishing whether diversity-dependence acts on extinction ($e^{D}\neq 0$), but not for estimating the precise value of the parameter. We do not believe that the difficulties estimating $e^{D}$ is primarily caused by shortcomings with the deep learning approach, as changing network architectures and training strategies did not improve the accuracy (results not shown). We suspect it is simply that extinction-related parameters are intrinsically difficult estimate across a range of biogeographical (Goldberg et al., 2011; Swiston and Landis, 2024) and diversity-dependent (Rabosky, 2009; Etienne et al., 2016) scenarios.

As a proof-of-concept, we apply our model to the empirical phylogenies and biogeographies of *Anolis* and *Viburnum*. For the *Anolis* system, our best-fitting model shows strong support for diversity-dependent within-region speciation and diversity-dependent extinction, but with weaker support for diversity-dependent incoming dispersal. Specifically, the best-fitting model supports the hypothesis where higher local species diversity reduces within-region speciation and incoming dispersal, and increases extinction events. Poe et al. (2017) previously noted that *Anolis* species experienced a relatively even number of dispersal events throughout time, which is consistent with the reduced support we estimated for diversity-dependent dispersal. In a separate study using a diversity-dependent MuSSE model, (Rabosky and Glor, 2010) found evidence of deterministically declining rates of diversification in Caribbean anoles (Rabosky and Glor, 2010), corroborating our estimates for diversity-dependent speciation and extinction. However, we caution readers that the precise value of $e^{D}$ parameter in Table 4 may not be reliable, and is better interpreted merely as $e^{D}>0$.

Applying our model to the empirical system of the *Oreinotinus* clade (*Viburnum*), we recovered strong support for a scenario where higher levels of regional species richness limit within-region speciation, and moderate support for diversity-dependent back-dispersal events into previously occupied regions, but found no clear support for diversity-dependent extinction. We think the support for diversity-dependent dispersal is significantly weaker than diversity-dependent within-region speciation in part because the tree is significantly smaller than the *Anolis* tree, and therefore contains less signal from the tips.

Furthermore, these results align with a scenario where each region is initially seeded by one or two colonizing lineages that diversified early-on until niche saturation prevented the further accumulation of species richness. This is supported by the observation that species richness is higher in the longer-occupied areas and lower in more recently occupied regions (Donoghue et al., 2022), but the difference is too small to be compatible with the unbounded, exponential growth implied by a standard GeoSSE model. We predict that the negative effect of resident species richness on dispersal as consistent with an incumbency effect: i.e., resident species are locally adapted and less likely to be dislodged by newcomers. The long, terminal branches of the *Oreinotinus* phylogeny are also characteristic of scenarios with decreasing extinction and speciation rates. Moreover, recent study documented a likely case of incipient speciation for *Oreinotinus* in the recently occupied Central Andes, indicating that perhaps the clade has not yet equilibrium diversity in the southern parts of its distribution (Maya-Lastra et al., 2024).

## Conclusion

We have described a novel birth-death process, called DDGeoSSE, that models how spatial rates of speciation, extinction, and dispersal depend on regional patterns of species richness. We derived key mathematical results from DDGeoSSE and explored the statistical properties of tree shapes generated under our model using simulations. We demonstrated the accuracy of parameter estimation under our model by applying a deep learning approach for phylogenetic studies. By applying our model to two empirical systems, we showed how it can be used to test a range of hypotheses in which local species diversity plays a major role in biogeographic radiation.

## Supplementary Material

Supplement 1

## Figures and Tables

**Table 1:** DDGeoSSE relationships between five biogeographic event types, biogeographic rates, and diversity-dependent effect parameters as regional species richness grows.

		Parameters		Effect on rate		

Process, p	Region(s)	Effect	Rate	$p^{D}<0$	$p^{D}=0$	$p^{D}>0$

Extinction	i	$e^{D}$	$e_{i}(t)$	↓	–	↑*
Within-region speciation	i	$w^{D}$	$w_{i}(t)$	↓*	–	↑
Dispersal (outbound)	i	$d^{D,\mathrm{src}}$	$d_{ij}(t)$	↓	–	↑
Dispersal (inbound)	i	$d^{D,\mathrm{dest}}$	$d_{ji}(t)$	↓*	–	↑
Between-region speciation	i∈k,j∈ℓ (ranges k and ℓ)	$b^{D}$	$b_{\ell }^{k}(t)$	↓	–	↑

Negative diversity-dependent effect values decrease the relevant event rates (↓), and positive effect values increase the rates (↑). Zero effect values correspond to the case where species diversity has no influence on event rates (−). Diversity-dependent effects that are usually associated with ecological competition are marked with asterices (*): larger numbers of species in region i are predicted to increase (↑) local extinction rates $\left(e_{i}(t)\right)$ and decrease (↓) local within-region speciation $\left(w_{i}(t)\right)$ and inbound dispersal ($d_{ji}(t$) rates.

**Table 2:** Settings for eight Log-DDG submodels without any diversity-dependent effects (0) and with one more more diversity-dependent effects (1–7).

Submodel	Extinction	Within-regior speciation	Dispersal

0	-	-	-
1	$e^{D}>0$	-	-
2	-	$w^{D}<0$	-
3	-	-	$d^{D,\mathrm{dest}}<0$
4	$e^{D}>0$	$w^{D}<0$	-
5	$e^{D}>0$	-	$d^{D,\mathrm{dest}}<0$
6	-	$w^{D}<0$	$d^{D,\mathrm{dest}}<0$
7	$e^{D}>0$	$w^{D}<0$	$d^{D,\mathrm{dest}}<0$

Entries marked with a dash (-) correspond to processes with no diversity-dependent effects.

**Table 3:** Table showing simulation conditions and priors distributions used to simulate training datasets for each submodel.

Submodel	Simulation condition	Min #taxa	Max #taxa	Max time	base rates ($\beta_{k},\beta_{k},\beta_{b},\beta_{b}$)	$w^{p}$	$e^{D}$	$d^{\mathrm{D,dest}}$	$d^{\mathrm{D,src}},\;b^{D}$
0	time & taxa count	10	U(100,300)	U(70,100)	{U(10^−4^,1), LogU(10^−4^,1)}	0	0	0	0
						0	U(10^−4^,2)	0	0
2						U(−2,−10^−4^)	0	0	0
3						0	0	U(−2,−10^−4^)	0
4						U(−2,−10^−4^)	U(10^−4^,2)	0	0
5						0	U(10^−4^,2)	U(−2,−10^−4^)	0
6						U(−2,−10^−4^)	0	u(−2,−10^−4^)	0
7						u(−2,−10^−4^)	U(10^−4^,2)	u(−2,−10^−4^)	0

**Table 4:** DDGeoSSE estimates for Caribbean *Anolis* lizards and Neotropical *Oreinotinus* plants using phyddle.

	Estimate	*Anolis*		*Viburnum*	

Parameter estimation for best-fitting model	$\rho_{w}$	0.2700	[0.1465, 0.5207]	1.1382	[0.4711,1.7549]
	$\rho_{e}$	0.0002	[0.0001,0.0012]	0.0021	[0.00105, 0.0175]
	$\rho_{d}$	0.0010	[0.0004, 0.0020]	0.0070	[0.00056, 0.0161]
	$\rho_{b}$	0.1036	[0.0016, 0.4191]	0.0028	[0.0007,0.2345]
	$w^{D}$	−1.64	[−1.86, −0.88]	−1.52	[−1.73,−1.09]
	$e^{D}$	0.71	[0.18,1.66]	−	−
	$d^{D,\mathrm{dest}}$	−1.59	[−1.93, −0.23]	−1.13	[−1.98, −0.26]

**Validation loss score**		0.609		0.478	

Rows 1–7 show point estimates and 80% CPIs (in brackets) for the best-fitting model. Rows 1–4 show base rates for within-region speciation $\left(\rho_{w}\right)$, extinction $\left(\rho_{e}\right)$, dispersal $\left(\rho_{d}\right)$, and between-region speciation $\left(\rho_{b}\right)$. Rows 5–7 show diversity-dependent effect parameters for within-region speciation ($w^{D}$), extinction ($e^{D}$), and incoming dispersal ($d^{D,\mathrm{dest}}$). Note that the base rates for *Viburnum* have been converted to correspond to the original phylogeny (branch lengths are measured in unit time). The last column shows the loss score calculated from each model’s corresponding validation dataset.

**Table 5:** DDGeoSSE estimates for Caribbean *Anolis* lizards and Neotropical *Oreinotinus* plants using phyddle.

Options	Network	*Anolis*			*Viburnum*			Validation Loss Score		

		$P\left(w^{D}\neq 0\right)$	$P\left(e^{D}\neq 0\right)$	$P\left(d^{D}\neq 0\right)$	$P\left(w^{D}\neq 0\right)$	$P\left(e^{D}\neq 0\right)$	$P\left(d^{D}\neq 0\right)$	$P\left(w^{D}\neq 0\right)$	$P\left(e^{D}\neq 0\right)$	$P\left(d^{D}\neq 0\right)$

Without weight-decay	Network 1	1.00	1.00	0.87	0.98	0.49	0.86	0.42	0.39	0.42
	Network 2	1.00	1.00	0.94	0.98	0.43	0.49	0.40	0.38	0.42
	Network 3	0.98	0.98	0.80	0.93	0.25	0.97	0.43	0.38	0.40
	Network 4	1.00	0.98	0.85	0.79	0.31	0.71	0.41	0.38	0.42

With weight-decay = 0.001	Network 5	0.97	0.83	0.97	0.97	0.29	0.75	0.38	0.37	0.37
	Network 6	0.99	0.95	0.80	0.89	0.43	0.61	0.39	0.35	0.39
	Network 7	0.97	0.94	0.97	0.89	0.51	0.55	0.39	0.36	0.37
	Network 8	0.96	0.79	0.94	0.91	0.42	0.67	0.38	0.36	0.37

**Mean value**		**0.98**	**0.93**	**0.89**	**0.92**	**0.39**	**0.70**	**0.40**	**0.37**	**0.39**

Networks 1–4 are trained without using non-zero weight_decay option, while networks 5–8 are trained using weight_decay of 0.001. Model selection support is reported as the marginal probability of the presence of diversity-dependent effects on within-region speciation $\left(P\left(w^{D}\neq 0\right)\right)$, extinction $\left(P\left(e^{D}\neq 0\right)\right)$, and incoming dispersal $\left(P\left(d^{D,\mathrm{dest}}\neq 0\right)\right)$ using 24 trained networks (8 for each evolutionary process). The last three columns show the loss scores from the validation dataset from each network for each process. The last row shows the average probabilities and validation loss scores across all networks.

## Data Availability

The dataset and all the relevant code used for this study are publicly available on https://github.com/alberts2/DDGeoSSE.
