# Supplementary material for: Phylogenetic estimation of diversity-dependent biogeographic rates using deep learning
Source: bioRxiv. 2026 Feb 18:2026.02.17.706216. Preprint. [Version 1] doi: 10.64898/2026.02.17.706216 (PMC12934830; doi:10.64898/2026.02.17.706216)
Supplement: Supplement 1 [file NIHPP2026.02.17.706216v1-supplement-1.pdf]

bioRxiv preprint doi: https://doi.org/10.64898/2026.02.17.706216; this version posted February 18, 2026. The copyright holder for this preprint (which was not certified by peer review) is the author/funder, who has granted bioRxiv a license to display the preprint in perpetuity. It is made available under aCC-BY 4.0 International license.

Supplementary Materials

GeoSSE events and state space

We describe a biogeographical model of species diversification, GeoSSE by Goldberg et al. (2011), which extends the state-dependent speciation and extinction (SSE) framework by associating species' biogeographical ranges with rates of evolution. The GeoSSE model is a joint framework that models the evolution of species range and both cladogenetic and anagenetic events simultaneously.

Anagenetic events in GeoSSE include species dispersal and local extinction (also known as extirpation) events. A dispersal event leads to range expansion where a species occupies an additional region that makes up its range. On the contrary, a local extinction event leads to range contraction where a species becomes locally extinct in one region. Moreover, a species becomes globally extinct once it goes locally extinct in the last region in its range. Note that like many other diversification models, GeoSSE also only allows one event occurring within an infinitesimal timestep. As a consequence of this assumption, a widespread species (i.e., species that occupies more than one region) cannot experience a complete extinction through a single event under the model.

Cladogenetic events in GeoSSE include within-region speciation and between-region speciation events. Each within-region speciation leads to creation of a new species within any single region of the parental species range. Each between-region speciation causes a widespread parental species and its range to split, such that all

bioRxiv preprint doi: https://doi.org/10.64898/2026.02.17.706216; this version posted February 18, 2026. The copyright holder for this preprint (which was not certified by peer review) is the author/funder, who has granted bioRxiv a license to display the preprint in perpetuity. It is made available under aCC-BY 4.0 International license.

regions in the parental range are distributed among the two new daughter species. Figure 6 illustrates how we assign rates to these different events and an example tree evolving under GeoSSE model.

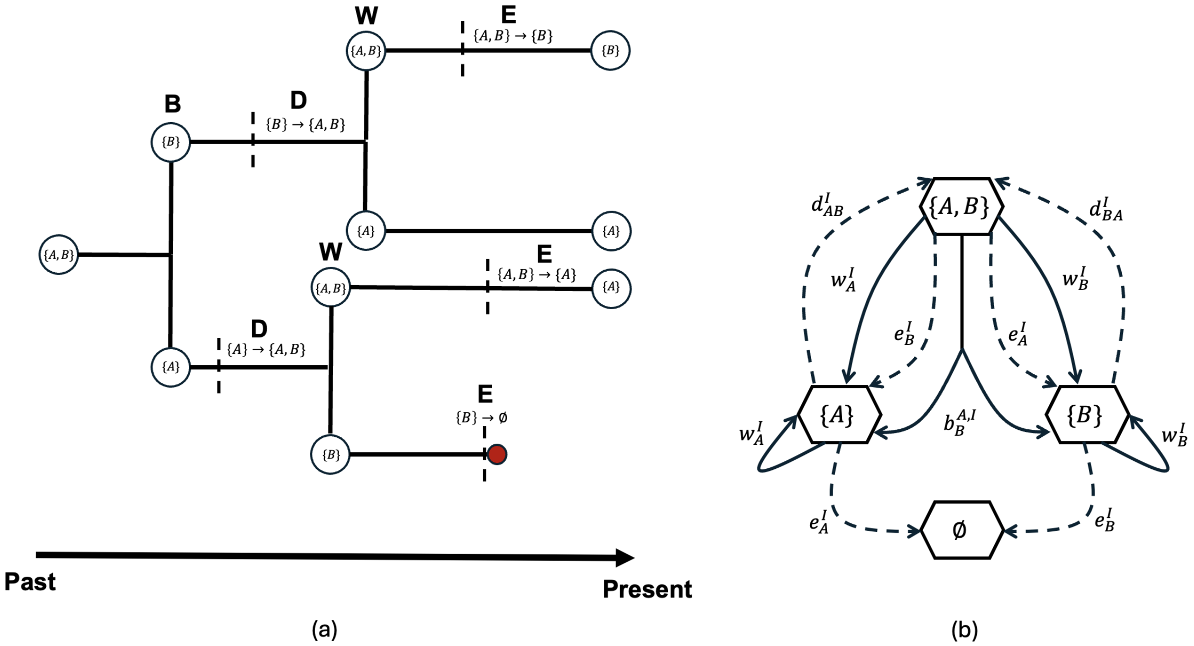

Figure 6: (a) Example phylogenetic tree showing all the history of four different GeoSSE event types in a two-region system (region A and B) of the model: within-region speciation event $\boldsymbol{W}$ associated with diversity-independent rates, w^I_A and w^I_B, between-region speciation event $\boldsymbol{B}$ associated with diversity-independent rate, $b^{A,I}_B$, range dispersal event $\boldsymbol{D}$ associated with diversity-independent rates, d^I_{AB} and d^I_{BA}, and extinction events (both local and global extinction of a species) $\boldsymbol{E}$ associated with diversity-independent rates, e^I_A and e^I_B. The red node represents a global extinction event of the corresponding species. (b) Transition diagram showing event types with their associated rates in a two-region GeoSSE system (GeoSSE model), following the structure in Goldberg et al. (2011). Solid arrows represent cladogenetic events and dashed arrows represent anagenetic events. Arrows going into the empty set represents a complete species extinction.

bioRxiv preprint doi: https://doi.org/10.64898/2026.02.17.706216; this version posted February 18, 2026. The copyright holder for this preprint (which was not certified by peer review) is the author/funder, who has granted bioRxiv a license to display the preprint in perpetuity. It is made available under aCC-BY 4.0 International license.

Incorporating diversity-independent rate factors

Here, we describe a way to incorporate diversity-independent rate factors for computing rates of evolution under our diversity-dependent framework. These rate factors can vary with respect to time, species ranges or regions. The absolute extinction rate in region $i \in \mathcal{R}$ can be described as follows:

$$\begin{aligned} e_i(t) &= \rho_e \times m_e(i,t), \\ m_e(i,t) &= m_e^I(i,t) \times m_e^D(i,t), \end{aligned} \tag{17}$$

where $m_e(i,t)$ is the relative rate factor for extinction process in region $i \in \mathcal{R}$ at time t. It is a product of the diversity-independent rate factor $m_e^I(i,t)$ and diversity-dependent rate factor $m_e^D(i,t)$, which is described in Eq. (2). Similarly, we can describe the absolute within-region speciation rate in region $i \in \mathcal{R}$ at time t as follows,

$$\begin{aligned} w_i(t) &= \rho_w \times m_w(i,t), \\ m_w(i,t) &= m_w^I(i,t) \times m_w^D(i,t), \end{aligned} \tag{18}$$

where $m_w(i,t)$ is the relative rate factor for within-region speciation process in region $i \in \mathcal{R}$ at time t. It is a product of the diversity-independent rate factor $m_w^I(i,t)$ and diversity-dependent rate factor $m_w^D(i,t)$, which is described in Eq. (4).

bioRxiv preprint doi: https://doi.org/10.64898/2026.02.17.706216; this version posted February 18, 2026. The copyright holder for this preprint (which was not certified by peer review) is the author/funder, who has granted bioRxiv a license to display the preprint in perpetuity. It is made available under aCC-BY 4.0 International license.

Next, we describe the absolute dispersal rate from region i to region j at time t as follows,

$$\begin{aligned} d_{ij}(t) &= \rho_d \times m_d(i,j,t), \\ m_d(i,j,t) &= m_d^I(i,j,t) \times m_d^D(i,j,t), \end{aligned} \tag{19}$$

where $m_d(i,j,t)$ is the relative rate factor for dispersal process from region i to region j at time t. It is a product of the diversity independent rate factor $m_d^I(i,j,t)$ and diversity-dependent rate factor $m_d^D(i,j,t)$, which is described in Eq. (6). Next, we describe the absolute between-region speciation rate, $b_\ell^k(t)$, that gives rise to two new species with ranges $k \in \mathcal{S}$ and $\ell \in \mathcal{S}$, respectively, from an ancestral species with range $m \in \mathcal{S}$ at time t as follows:

$$\begin{aligned} b_\ell^k(t) &= \rho_b \times m_b(k,\ell,t), \\ m_b(k,\ell,t) &= m_b^I(k,\ell,t) \times f_b(k,\ell,t;m_b^D), \end{aligned} \tag{20}$$

where $m_b(k,\ell,t)$ is the relative rate factor for between-region speciation process that gives rise to two new species with range k and ℓ. It is a product of the diversity-independent rate factor $m_b^I(k,\ell,t)$ and diversity-dependent rate factor m_b^D defined via the range split score function, $f_b(k,\ell,t;m_b^D)$, as described in Eq. (8). Note, the

bioRxiv preprint doi: https://doi.org/10.64898/2026.02.17.706216; this version posted February 18, 2026. The copyright holder for this preprint (which was not certified by peer review) is the author/funder, who has granted bioRxiv a license to display the preprint in perpetuity. It is made available under aCC-BY 4.0 International license.

definition of $m_b^I(k,\ell,t)$ is arbitrary. One could define $m_b^I(k,\ell,t)$ as $f_b(k,\ell,t;\dot{m}_b^I)$ where $\dot{m}_b^I(i,j,t)$ defines how all features shared between regions i and j influence the rate factors in the range split score function, f_b.

Model variant: Standard DDGeoSSE model

The Standard DDGeoSSE (Standard-DDG) model variant is similar to the Log-DDG model described in the main text, except diversity-dependence of Standard-DDG depends on the actual number of species in a region, rather than the actual natural log number of species. Regardless of which process (p) is considered, and unless otherwise stated, the variables in Equations 21 to 24 all share the similar constraints: species counts are positive ($n_i(t) > 0, n_j(t) > 0$), rates are positive ($p_i(t) > 0, \rho_p > 0$), relative rate factors are positive ($m_p^I(i,t) > 0, m_p^D(i,t) > 0, m_p^I(i,j,t) > 0, m_p^I(i,j,t) > 0$), and diversity-dependent effect parameters are real-valued ($\infty < \hat{p}^D < \infty$). We define the absolute extinction rate for a species in region i at time t as follows

$$\begin{aligned} e_i(t) &= \rho_e \times \hat{m}_e(i,t), \\ \hat{m}_e(i,t) &= m_e^I(i,t) \times \hat{m}_e^D(i,t), \\ \hat{m}_e^D(i,t) &= n_i(t)^{\hat{e}^D}, \end{aligned} \tag{21}$$

where the diversity-dependent effect parameter $\hat{e}^D$ has the same interpretations as e^D in Table 1. Additionally, in the case of $\hat{e}^D = 1$, we have a linear-like behaviour in overall rate increases (resp. decreases) over time as the number of species in region i increases (resp. decreases).

bioRxiv preprint doi: https://doi.org/10.64898/2026.02.17.706216; this version posted February 18, 2026. The copyright holder for this preprint (which was not certified by peer review) is the author/funder, who has granted bioRxiv a license to display the preprint in perpetuity. It is made available under aCC-BY 4.0 International license.

Similarly, we define the absolute within-region speciation rate for a species in region i at time t as follows

$$\begin{aligned} w_i(t) &= \rho_w \times \hat{m}_w(i,t), \\ \hat{m}_w(i,t) &= m_w^I(i,t) \times \hat{m}_w^D(i,t), \\ \hat{m}_w^D(i,t) &= n_i(t)^{\hat{w}^D}, \end{aligned} \tag{22}$$

where the diversity-dependent effect parameter $\hat{w}^D$ has the same interpretations as w^D in Table 1. Additionally, in the case of $\hat{w}^D = 1$, we have a linear-like behaviour in overall rate increases (resp. decreases) over time as the number of species in region i increases (resp. decreases).

Next, we define the absolute dispersal rate from region i to region j at time t, as follows,

$$\begin{aligned} d_{ij}(t) &= \rho_d \times \hat{m}_d(i,j,t), \\ \hat{m}_d(i,j,t) &= m_d^I(i,j,t) \times \hat{m}_d^D(i,j,t), \\ \hat{m}_d^D(i,j,t) &= n_i(t)^{\hat{d}^{D,src}}(n_j(t)+1)^{\hat{d}^{D,dest}} \end{aligned} \tag{23}$$

where the diversity-dependent effect parameters $\hat{d}^{D,src}$ and $\hat{d}^{D,dest}$ have the same interpretations as $d^{D,src}$ and $d^{D,dest}$, respectively, in Table 1. For dispersal, the destination region j may contain 0 species: $n_j(t) \geq 0$. Additionally, in the case of $\hat{d}^{D,src} = 1, \hat{d}^{D,dest} = 0$ or $\hat{d}^{D,src} = 0, \hat{d}^{D,dest} = 1$, we have a linear-like behavior in overall rate increases (resp. decreases) over time as the numbers of species in regions

bioRxiv preprint doi: https://doi.org/10.64898/2026.02.17.706216; this version posted February 18, 2026. The copyright holder for this preprint (which was not certified by peer review) is the author/funder, who has granted bioRxiv a license to display the preprint in perpetuity. It is made available under aCC-BY 4.0 International license.

i and j (both) increase (resp. decrease).

Finally, we define the absolute between-region speciation rate that gives rise to two new species with ranges $k \in \mathcal{S}$ and $\ell \in \mathcal{S}$, respectively, from an ancestral species with range $m \in \mathcal{S}$ at time t. We define the overall rate as follows:

$$
\begin{aligned}
b_\ell^k(t) &= \rho_b \times \hat{m}_b(k,\ell,t), \\
\hat{m}_b(k,\ell,t) &= m_b^I(k,\ell,t) \times f_b(k,\ell,t;\hat{m}_b^D), \\
f_b(k,\ell,t;\hat{m}_b^D) &\propto \left[\sum_{(i,j)\in E(k,\ell)} \left[\frac{\hat{m}_b^D(i,j,t) + \hat{m}_b^D(j,i,t)}{2} \right]^{-1} \right]^{-1}, \\
\hat{m}_b^D(i,j,t) &= (n_i(t) + n_j(t) - 1)^{\hat{b}^D} \qquad (24)
\end{aligned}
$$

where the diversity-dependent effect parameter $\hat{b}^D$ has the same interpretations as b^D in Table 1. Additionally, in the case of $\hat{b}^D = 1$, we have a linear-like behavior in absolute rate increases (resp. decreases) over time as the number of species in region i increases (resp. decreases).

Local equilibrium diversity under DDG with only diversity-dependent within-region speciation and extinction (no dispersal)

We consider a scenario where only diversity-dependent within-region speciation and extinction, but with no dispersal whatsoever, drive the diversification process. We derive the solution to the local equilibrium diversity described in the main text under this particular scenario.

Lemma 4. *Given the balance equation described in Eq.* (11), *the local equilibrium*

bioRxiv preprint doi: https://doi.org/10.64898/2026.02.17.706216; this version posted February 18, 2026. The copyright holder for this preprint (which was not certified by peer review) is the author/funder, who has granted bioRxiv a license to display the preprint in perpetuity. It is made available under aCC-BY 4.0 International license.

diversity in region i under scenario with only within-region process and constant extinction rate is given by

$$n_i^* = \exp\left\{\left(\frac{\rho_e}{\rho_w}\right)^{\frac{1}{w^D - e^D}} - 1\right\},\ w^D \neq e^D; \rho_e \neq 0 \text{ and } \rho_w \neq 0, \tag{25}$$

under the Log-DDG model, and

$$n_i^* = \left(\frac{\rho_e}{\rho_w}\right)^{\frac{1}{\hat{w}^D - \hat{e}^D}},\ \hat{w}^D \neq \hat{e}^D; \rho_e \neq 0 \text{ and } \rho_w \neq 0, \tag{26}$$

under the Standard-DDG model. Moreover, if we have constant extinction rate, then both Eqs. (25) *and* (26) *become*

$$n_i^* = \exp\left\{\left(\frac{\rho_e}{\rho_w}\right)^{\frac{1}{w^D}} - 1\right\},\ w^D \neq 0; \rho_e \neq 0 \text{ and } \rho_w \neq 0, \tag{27}$$

and

$$n_i^* = \left(\frac{\rho_e}{\rho_w}\right)^{\frac{1}{\hat{w}^D}},\ \hat{w}^D \neq 0; \rho_e \neq 0 \text{ and } \rho_w \neq 0, \tag{28}$$

respectively.

Proof. We show the proof for the lemma under the Log-DDG model as the proof under the Standard-DDG model follows in a similar manner.

bioRxiv preprint doi: https://doi.org/10.64898/2026.02.17.706216; this version posted February 18, 2026. The copyright holder for this preprint (which was not certified by peer review) is the author/funder, who has granted bioRxiv a license to display the preprint in perpetuity. It is made available under aCC-BY 4.0 International license.

Following Eq. (11) in Definition 1 and by substituting Eqs. (2) and (4) we have,

$$\begin{aligned} w_i(t) &= e_i(t) \\ \rho_w \left[\log(n_i(t)) + 1\right]^{w^D} &= \rho_e \left[\log(n_i(t)) + 1\right]^{e^D} \\ \left[\log(n_i(t)) + 1\right]^{w^D - e^D} &= \frac{\rho_e}{\rho_w} \\ n_i(t) &= \exp\left\{\left(\frac{\rho_e}{\rho_w}\right)^{\frac{1}{w^D - e^D}} - 1\right\}. \end{aligned} \tag{29}$$

So,

$$\begin{aligned} n_i^* &= \lim_{t\to\infty} n_i(t) \\ &= \exp\left\{\left(\frac{\rho_e}{\rho_w}\right)^{\frac{1}{w^D - e^D}} - 1\right\}. \end{aligned} \tag{30}$$

Then, we can set $e^D = 0$ in Eq. (30) to get a constant extinction rate. Clearly, ρ_w cannot be equal to 0. Moreover, given $\rho_w \neq 0$, ρ_e also cannot be equal to 0 since we assume that extinction process exists. □

Local equilibrium diversity under DDG with diversity-dependent within-region speciation

We derive the solution to the local equilibrium diversity described in the main text under Log-DDG model with diversity-dependent within-region speciation and time-constant extinction and dispersal.

Lemma 5. *Given the balance equation described in Eq.* (11)*, the local equilibrium diversity in region i under the DDGeoSSE model with diversity-dependent within-*

bioRxiv preprint doi: https://doi.org/10.64898/2026.02.17.706216; this version posted February 18, 2026. The copyright holder for this preprint (which was not certified by peer review) is the author/funder, who has granted bioRxiv a license to display the preprint in perpetuity. It is made available under aCC-BY 4.0 International license.

region speciation is given by,

$$n_i^* = \exp\left\{\left[\frac{\rho_e - \rho_d(|\mathcal{R}| - 1)}{\rho_w}\right]^{\frac{1}{w^D}} - 1\right\},\ w^D \neq 0; \rho_w \neq 0, \tag{31}$$

assuming $n_i^* \gg 0$ under the Log-DDG model.

Proof. Following Eq. (11) in Definition 1 and by substituting Eqs. (2) and (4) we have,

$$\begin{aligned} w_i(t) + \sum_{\substack{j \in R \\ j \neq i}} d_{ji} &= e_i(t) \\ \rho_w(\log(n_i(t)) + 1)^{w^D} + \rho_d(\log(n_i(t) + 1) + 1)^{d^{D,dest}} \sum_{\substack{j \in R \\ j \neq i}} (\log(n_j(t)) + 1)^{d^{D,src}} &= \rho_e(\log(n_i(t)) + 1)^{e^D} \end{aligned} \tag{32}$$

We solve the above equation at equilibrium diversity across locations ($t \to \infty$). That is,

$$\rho_w(\log(n_i^*) + 1)^{w^D} + \rho_d(\log(n_i^*) + 1)^{d^{D,dest}} \sum_{\substack{j \in R \\ j \neq i}} (\log(n_j^*) + 1)^{d^{D,src}} = \rho_e(\log(n_i^*) + 1)^{e^D}.$$

Then by assumption we have $d^{D,src} = d^{D,dest} = e^D = 0$. That is,

$$\rho_w(\log(n_i^*) + 1)^{w^D} + \rho_d(|\mathcal{R}| - 1) = \rho_e.$$

bioRxiv preprint doi: https://doi.org/10.64898/2026.02.17.706216; this version posted February 18, 2026. The copyright holder for this preprint (which was not certified by peer review) is the author/funder, who has granted bioRxiv a license to display the preprint in perpetuity. It is made available under aCC-BY 4.0 International license.

Thus,

$$n_i^* = \exp\left\{\left[\frac{\rho_e - \rho_d(|\mathcal{R}|-1)}{\rho_w}\right]^{\frac{1}{w^D}} - 1\right\},\ w^D \neq 0; \rho_w \neq 0.$$

□

Local equilibrium diversity under DDG with diversity-dependent extinction

We derive the solution to the local equilibrium diversity described in the main text under Log-DDG model with diversity-dependent extinction and time-constant within-region speciation and dispersal.

Lemma 6. *Given the balance equation described in Eq.* (11)*, the local equilibrium diversity in region i under DDGeoSSE model with diversity-dependent extinction is given by,*

$$n_i^* = \exp\left\{\left[\frac{\rho_w + \rho_d(|\mathcal{R}|-1)}{\rho_e}\right]^{\frac{1}{e^D}} - 1\right\},\ e^D \neq 0; \rho_e \neq 0, \tag{33}$$

assuming $n_i^* \gg 0$ under the Log-DDG model.

Proof. Following the same steps as the proof for Lemma 5 and by substituting $d^{D,src} = d^{D,dest} = w^D = 0$ instead, it follows that

$$n_i^* = \exp\left\{\left[\frac{\rho_w + \rho_d(|\mathcal{R}|-1)}{\rho_e}\right]^{\frac{1}{e^D}} - 1\right\},\ e^D \neq 0; \rho_e \neq 0.$$

□

We demonstrate Lemma 6 using simulations, as shown in Figure 7

bioRxiv preprint doi: https://doi.org/10.64898/2026.02.17.706216; this version posted February 18, 2026. The copyright holder for this preprint (which was not certified by peer review) is the author/funder, who has granted bioRxiv a license to display the preprint in perpetuity. It is made available under aCC-BY 4.0 International license.

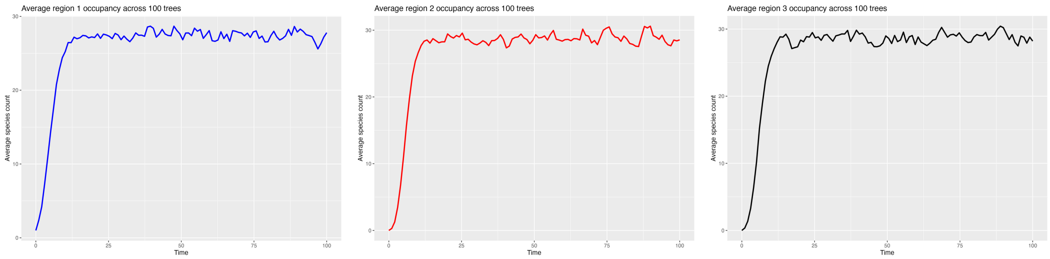

Figure 7: The trajectories of $n_i(t)$ in all three regions where 100 trees are simulated the Log-DDG model with $n_i^* = 30$, $\rho_w = 1.0$, $\rho_e = 0.03$, $\rho_d = 0.1$, $\rho_b = 0$, $w^D = d^{D,src} = d^{D,dest} = b^D = 0$, and e^D was chosen according to Lemma 6. For each tree simulation, we initialize a tree with root species in range $\{A\}$.

Proof of Lemma 2

Proof. Following Eq. (11) in Definition 1 and by substituting Eqs. (2) and (4) we have,

$$\begin{aligned} w_i(t) + \sum_{\substack{j \in R \\ j \neq i}} d_{ji} &= e_i(t) \\ \rho_w(\log(n_i(t)) + 1)^{w^D} + \rho_d(\log(n_i(t) + 1) + 1)^{d^{D,dest}} \sum_{\substack{j \in R \\ j \neq i}} (\log(n_j(t)) + 1)^{d^{D,src}} &= \rho_e(\log(n_i(t)) + 1)^{e^D}. \end{aligned} \tag{34}$$

We solve the above equation at equilibrium diversity across locations ($t \to \infty$). That is,

$$\rho_w(\log(n_i^*) + 1)^{w^D} + \rho_d(\log(n_i^*) + 1)^{d^{D,dest}} \sum_{\substack{j \in R \\ j \neq i}} (\log(n_j^*) + 1)^{d^{D,src}} = \rho_e(\log(n_i^*) + 1)^{e^D}.$$

bioRxiv preprint doi: https://doi.org/10.64898/2026.02.17.706216; this version posted February 18, 2026. The copyright holder for this preprint (which was not certified by peer review) is the author/funder, who has granted bioRxiv a license to display the preprint in perpetuity. It is made available under aCC-BY 4.0 International license.

Assuming $n_i^* \gg 0$, $w^D = d^{D,dest} = y$, and $d^{D,src} = 0$, we have,

$$\begin{aligned}\rho_w(\log(n_i^*)+1)^y + \rho_d(\log(n_i^*)+1)^y(|\mathcal{R}|-1) &= \rho_e(\log(n_i^*)+1)^{e^D} \\ (\log(n_i^*)+1)^{(e^D-y)} &= \frac{\rho_w + \rho_d(|\mathcal{R}-1|)}{\rho_e}.\end{aligned}$$

Thus,

$$n_i^* = \exp\left\{\left[\frac{\rho_w + \rho_d(|\mathcal{R}|-1)}{\rho_e}\right]^{\frac{1}{e^D-y}} - 1\right\},\ e^D \neq y; \rho_e \neq 0.$$

□

Proof of Lemma 3

Proof. Following Eq. (11) in Definition 1 and by substituting Eqs. (2) and (4) we have,

$$\begin{aligned}w_i(t) + \sum_{\substack{j\in R\\ j\neq i}} d_{ji} &= e_i(t) \\ \rho_w(\log(n_i(t))+1)^{w^D} + \rho_d(\log(n_i(t)+1)+1)^{d^{D,dest}} \sum_{\substack{j\in R\\ j\neq i}} (\log(n_j(t))+1)^{d^{D,src}} &= \rho_e(\log(n_i(t))+1)^{e^D}.\end{aligned} \tag{35}$$

We solve the above equation at equilibrium diversity across locations ($t \to \infty$). That

bioRxiv preprint doi: https://doi.org/10.64898/2026.02.17.706216; this version posted February 18, 2026. The copyright holder for this preprint (which was not certified by peer review) is the author/funder, who has granted bioRxiv a license to display the preprint in perpetuity. It is made available under aCC-BY 4.0 International license.

is,

$$\rho_w(\log(n_i^*)+1)^{w^D} + \rho_d(\log(n_i^*)+1)^{d^{D,dest}} \sum_{\substack{j\in R \\ j\neq i}} (\log(n_j^*)+1)^{d^{D,src}} \;=\; \rho_e(\log(n_i^*)+1)^{e^D}.$$

Assuming $n_i^* \gg 0$ and $w^D = d^{D,dest} = d^{D,src} = x$, we have

$$\rho_w(\log(n_i^*)+1)^x + \rho_d(\log(n_i^*)+1)^x(|\mathcal{R}|-1)(\log(n_j^*)+1)^x \;=\; \rho_e(\log(n_i^*)+1)^{e^D}.$$

Note here from model assumptions, $n_i^* = n_j^*, \forall j \neq i$. Then,

$$n_i^* = \exp\left\{ \left[\frac{\rho_w + \rho_d(|\mathcal{R}|-1)(\log(n_j^*)+1)^x}{\rho_e} \right]^{\frac{1}{e^D - x}} - 1 \right\},\ \rho_e \neq 0. \tag{36}$$

□

Local equilibrium diversity under Standard-DDG with full process and without diversity-dependent on outbound dispersal

Lemma 7. *Given the balance equation described in Eq.* (11) *and* $|\mathcal{R}|$ *denotes the number of discrete regions in the system, the local equilibrium diversity in region* i *under the full process with no diversity-dependent effect on dispersal in the source region,* $(d^{D,src} = 0)$, *is given by*

$$n_i^* = \left[\frac{\rho_w + \rho_d(|\mathcal{R}|-1)}{\rho_e} \right]^{\frac{1}{\hat{e}^D - \hat{y}}},\ \hat{e}^D \neq \hat{y}; \rho_e \neq 0, \tag{37}$$

bioRxiv preprint doi: https://doi.org/10.64898/2026.02.17.706216; this version posted February 18, 2026. The copyright holder for this preprint (which was not certified by peer review) is the author/funder, who has granted bioRxiv a license to display the preprint in perpetuity. It is made available under aCC-BY 4.0 International license.

assuming $n_i^* \gg 0$ *and* $\hat{w}^D = \hat{d}^{D,dest} = \hat{y}$ *under the Standard-DDG model.*

Proof. The proof under the Standard-DDG model follows in a similar manner to the proof under the Log-DDG model in Lemma 2. □

Local equilibrium diversity under Standard-DDG with full process and time-varying outbound dispersal

Lemma 8. *Given the balance equation described in Eq.* (11) *and* $|\mathcal{R}|$ *denotes the number of discrete regions in the system, the local equilibrium diversity in region* i *under the full process with time-varying dispersal rate into region* i *is given by*

$$n_i^* = \left[\frac{\rho_w + \rho_d(|\mathcal{R}| - 1)(n_j^*)^{\hat{x}}}{\rho_e}\right]^{\frac{1}{\hat{e}^D - \hat{x}}}, \ \hat{e}^D \neq \hat{x}; \rho_e \neq 0, \tag{38}$$

assuming $n_i^* \gg 0$ *and* $\hat{w}^D = \hat{d}^{D,dest} = \hat{d}^{D,src} = \hat{x}$ *under the Standard-DDG model. Note* $n_j^* = n_i^*, \forall j \neq i$ *since the model assumes equal local equilibrium diversity between* i *and* j.

Proof. The proof under the Standard-DDG model follows in a similar manner to the proof under the Log-DDG model in Lemma 3. □

Tree statistics near the extreme case

bioRxiv preprint doi: https://doi.org/10.64898/2026.02.17.706216; this version posted February 18, 2026. The copyright holder for this preprint (which was not certified by peer review) is the author/funder, who has granted bioRxiv a license to display the preprint in perpetuity. It is made available under aCC-BY 4.0 International license.

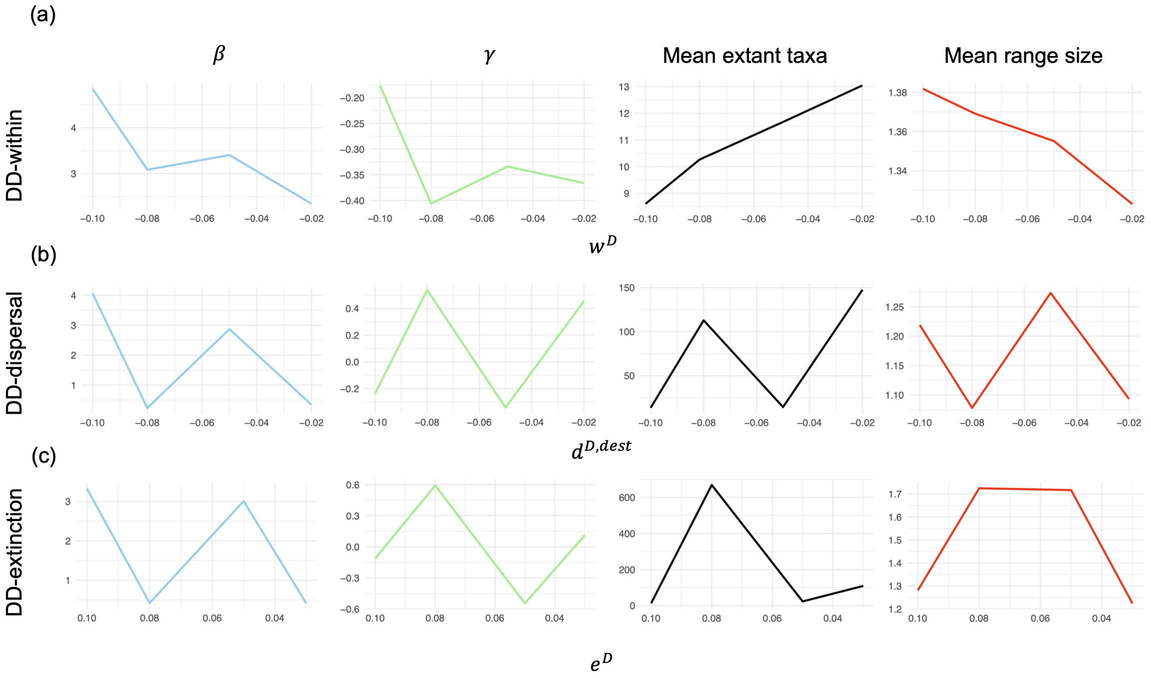

Figure 8: Plots showing changes in the values of various tree statistics from simulated trees drawn from model with diversity-dependent effect on (a) within-region speciation ("DD-within"), (b) dispersal ("DD-dispersal"), and (c) extinction ("DD-extinction") under the extreme case scenarios for the effect of diversity-dependence on rates. The $x-$axis represents the values of each diversity-dependent rate scalar respectively $\left(w^D, d^{D,dest}, e^D\right)$ and the $y-$axis represent the values of the tree statistics.

Here, we consider at a case where our Log-DDG model closely resembles the standard GeoSSE model. This can be achieved by simulating trees under the condition where the diversity-dependent effects have their values drawn from intervals near 0 $\left(w^D, e^D, d^{D,dest} \approx 0\right)$.

As seen from Figure 8(a), some statistics, such as the β statistic (Fig. 8(a), first column), mean range size (Fig. 8(a), fourth column), and mean number of extant taxa (Fig. 8, third column), have similar trend to those from Figure 4(a). However,

bioRxiv preprint doi: https://doi.org/10.64898/2026.02.17.706216; this version posted February 18, 2026. The copyright holder for this preprint (which was not certified by peer review) is the author/funder, who has granted bioRxiv a license to display the preprint in perpetuity. It is made available under aCC-BY 4.0 International license.

as expected, the variability in their values is less noticeable compared to those from Figure 4(a) due to w^D having values near 0.

As for Figure 8(c), it is noticeably harder to identify the trend of changes in these statistics values, possibly due to all e^D having values near 0. However, notice that both β and γ values seem to agree when $w^D \to 0^-$ from negative real axis and $e^D \to 0^+$ from positive real axis (Figs. 8(a) & 8(c), first and second columns).

Similar to Figure 8(c), it is also noticeably harder to identify trend for change in values on these statistics for the model with only diversity-dependent effect on incoming dispersal (Fig. 8(b)), possibly due to $d^{D,dest}$ has all its values drawn near 0.

Tree shape under diversity-dependent within-region speciation and extinction

Here, we study tree shape statistics for trees simulated under our model with diversity-dependent effect on both within-region speciation and extinction. In summary, we observe consistent trends in the tree statistics when compared those using trees simulated under a single diversity-dependent effect on either processes. That is, the average range size is at the highest when $w^D \ll 0$ and $e^D \approx 0$ (Fig. 9, bottom left panel), and the average number of extant taxa is at the maximum when both $w^D, e^D \approx 0$ (Fig. 9, bottom right panel). Furthermore, consistent trends are also observed for both β and γ statistics, namely β values tend to be larger when $w^D \ll 0$ and $e^D \gg 0$ (Fig. 9, top left panel), and γ values tend to be larger when both $w^D, e^D \approx 0$ (Fig. 9, top right panel).

Tree shape under diversity-dependent incoming dispersal and extinction

bioRxiv preprint doi: https://doi.org/10.64898/2026.02.17.706216; this version posted February 18, 2026. The copyright holder for this preprint (which was not certified by peer review) is the author/funder, who has granted bioRxiv a license to display the preprint in perpetuity. It is made available under aCC-BY 4.0 International license.

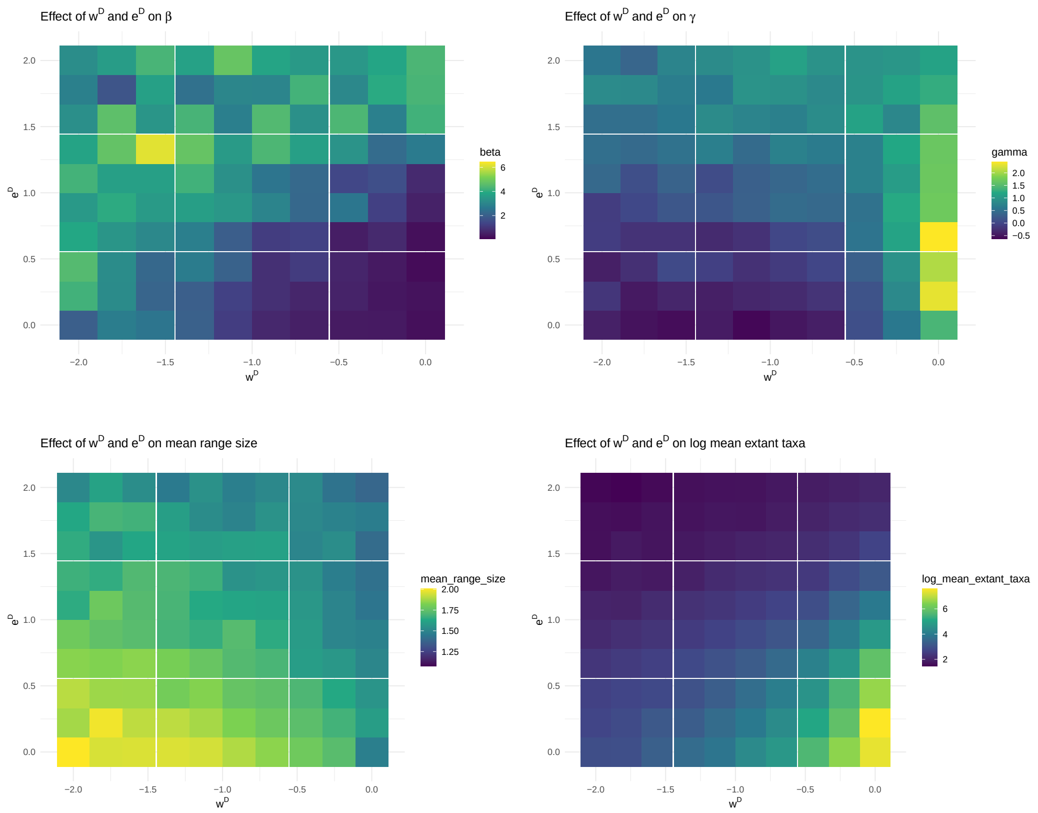

Figure 9: Various heatmaps showing changes in various tree statistics values due to varying degree of diversity-dependent effects on both within-region speciation and extinction.

Here, we study tree shape statistics for trees simulated under our model with diversity-dependent effect on both incoming dispersal and extinction. In summary, we observe the similar trend on both the average range size and average number of extant taxa when combining diversity-dependent effect on both extinction and incoming dispersal. That is, we observe higher range size and number of taxa in the region where the effect is weaker on both events (Fig. 10).

bioRxiv preprint doi: https://doi.org/10.64898/2026.02.17.706216; this version posted February 18, 2026. The copyright holder for this preprint (which was not certified by peer review) is the author/funder, who has granted bioRxiv a license to display the preprint in perpetuity. It is made available under aCC-BY 4.0 International license.

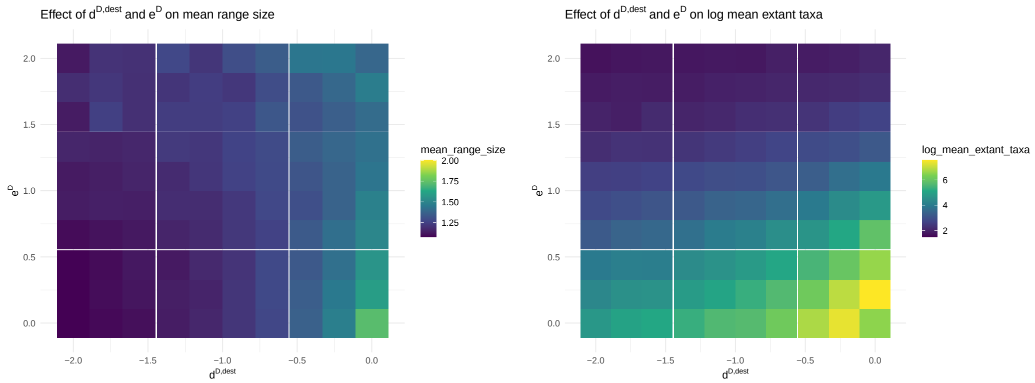

Figure 10: Various heatmaps showing changes in mean range size and mean number of extant taxa (in logarithmic scale) due to varying degree of diversity-dependent effects on both incoming dispersal and extinction.

Performance of parameter estimation using Submodel 0

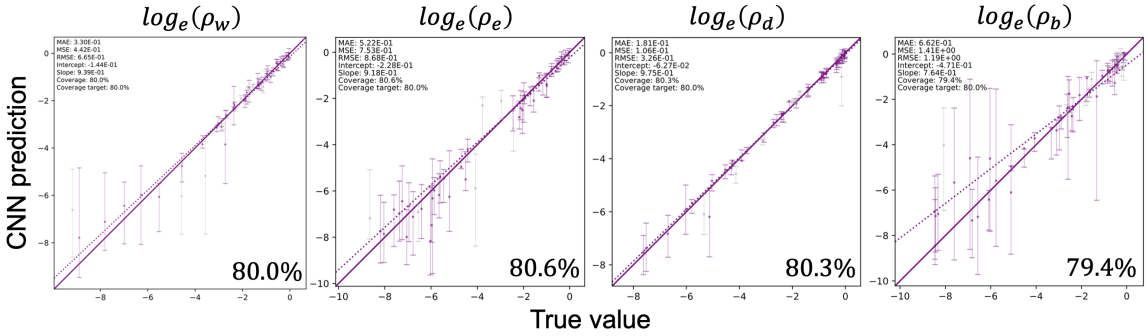

Figure 11: Plots showing 80% CPIs for each parameter on the test dataset simulated under submodel 0. The $x-$axis is showing the true parameter values, and the $y-$axis is showing the estimated values from CNN. All test data were used for the regression. Of these, point estimates (markers) and 80% CPIs (bars) are shown for 50 examples.

bioRxiv preprint doi: https://doi.org/10.64898/2026.02.17.706216; this version posted February 18, 2026. The copyright holder for this preprint (which was not certified by peer review) is the author/funder, who has granted bioRxiv a license to display the preprint in perpetuity. It is made available under aCC-BY 4.0 International license.

Performance of parameter estimation using Submodel 1

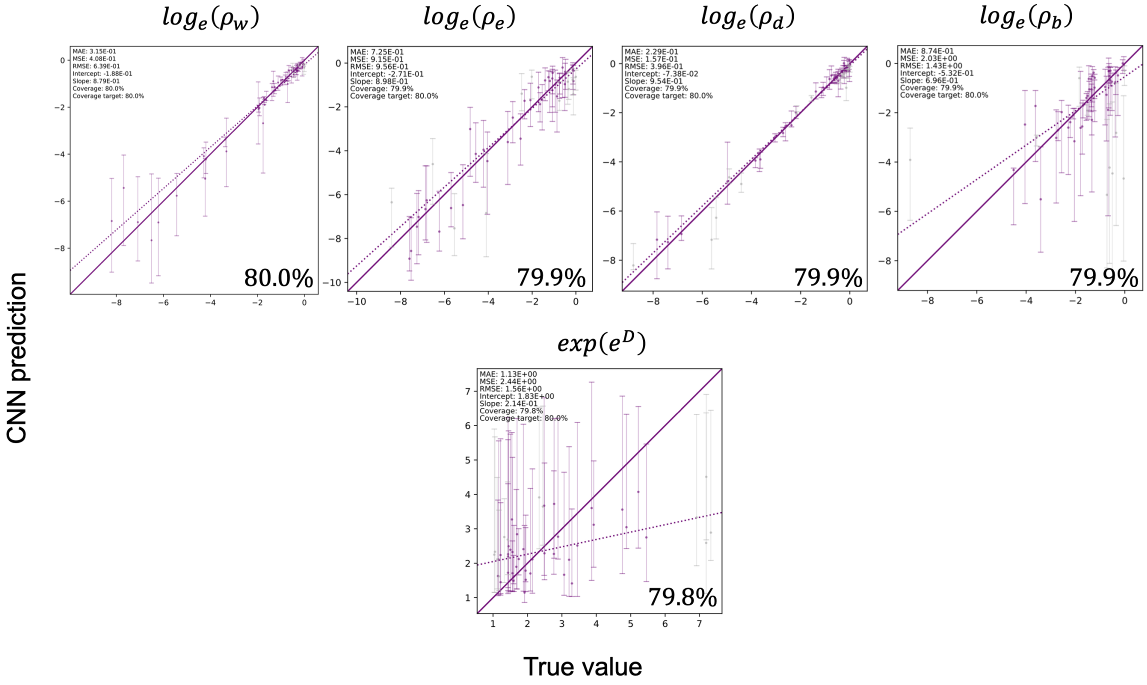

Figure 12: Plots showing 80% CPIs for each parameter on the test dataset simulated under submodel 1. The $x-$axis is showing the true parameter values, and the $y-$axis is showing the estimated values from CNN. All test data were used for the regression. Of these, point estimates (markers) and 80% CPIs (bars) are shown for 50 examples.

bioRxiv preprint doi: https://doi.org/10.64898/2026.02.17.706216; this version posted February 18, 2026. The copyright holder for this preprint (which was not certified by peer review) is the author/funder, who has granted bioRxiv a license to display the preprint in perpetuity. It is made available under aCC-BY 4.0 International license.

1085 *Performance of parameter estimation using Submodel 2*

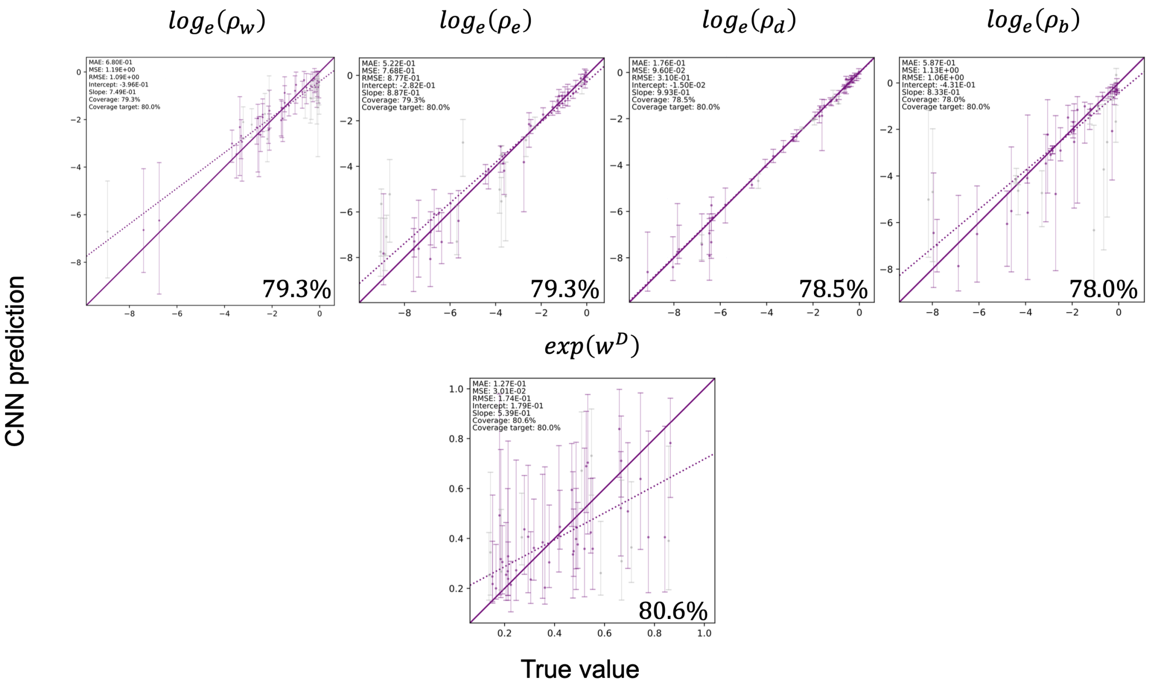

Figure 13: Plots showing 80% CPIs for each parameter on the test dataset simulated under submodel 2. The $x-$axis is showing the true parameter values, and the $y-$axis is showing the estimated values from CNN. All test data were used for the regression. Of these, point estimates (markers) and 80% CPIs (bars) are shown for 50 examples.

bioRxiv preprint doi: https://doi.org/10.64898/2026.02.17.706216; this version posted February 18, 2026. The copyright holder for this preprint (which was not certified by peer review) is the author/funder, who has granted bioRxiv a license to display the preprint in perpetuity. It is made available under aCC-BY 4.0 International license.

Performance of parameter estimation using Submodel 3

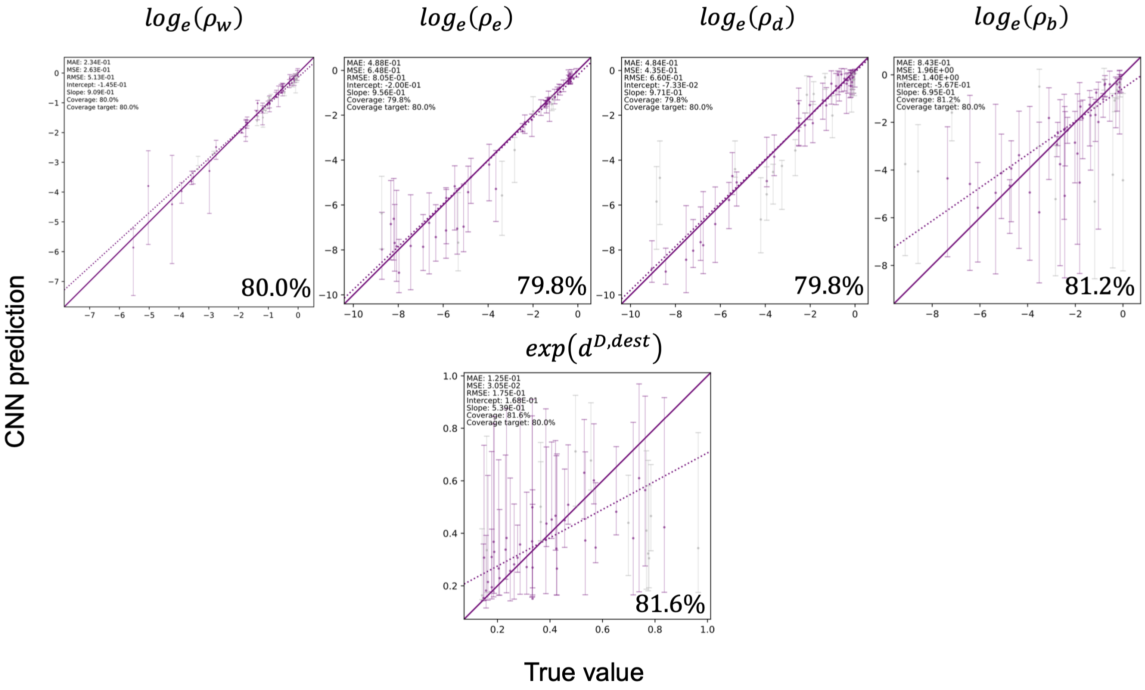

Figure 14: Plots showing 80% CPIs for each parameter on the test dataset simulated under submodel 3. The $x-$axis is showing the true parameter values, and the $y-$axis is showing the estimated values from CNN. All test data were used for the regression. Of these, point estimates (markers) and 80% CPIs (bars) are shown for 50 examples.

bioRxiv preprint doi: https://doi.org/10.64898/2026.02.17.706216; this version posted February 18, 2026. The copyright holder for this preprint (which was not certified by peer review) is the author/funder, who has granted bioRxiv a license to display the preprint in perpetuity. It is made available under aCC-BY 4.0 International license.

Performance of parameter estimation using Submodel 4

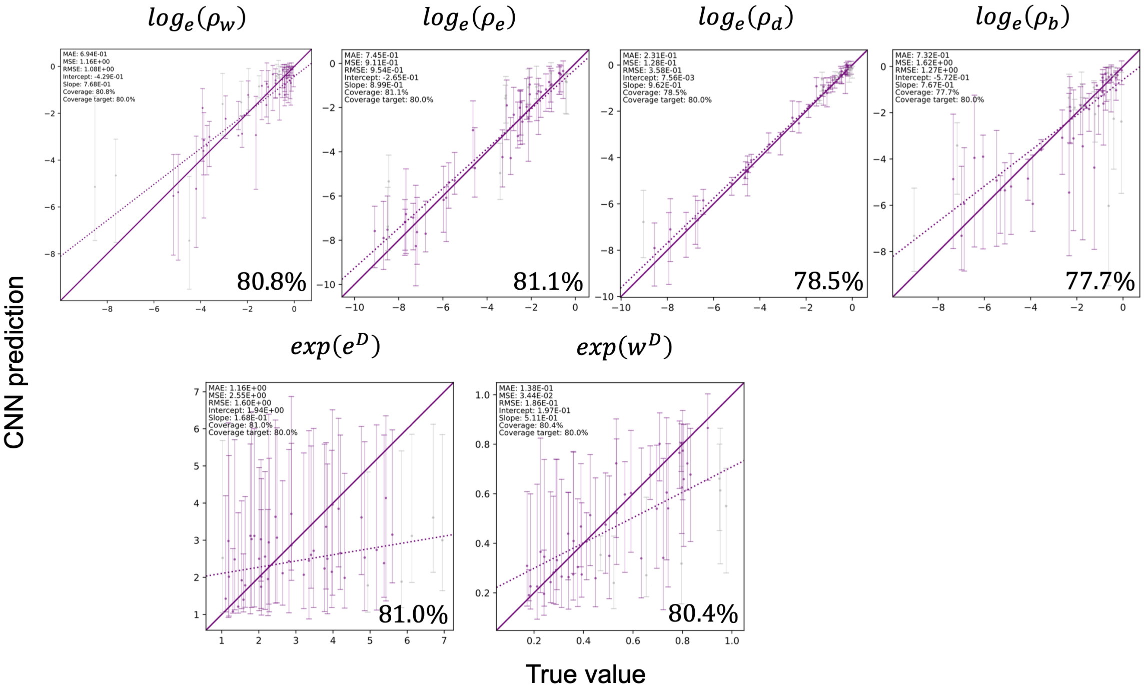

Figure 15: Plots showing 80% CPIs for each parameter on the test dataset simulated under submodel 4. The $x-$axis is showing the true parameter values, and the $y-$axis is showing the estimated values from CNN. All test data were used for the regression. Of these, point estimates (markers) and 80% CPIs (bars) are shown for 50 examples.

bioRxiv preprint doi: https://doi.org/10.64898/2026.02.17.706216; this version posted February 18, 2026. The copyright holder for this preprint (which was not certified by peer review) is the author/funder, who has granted bioRxiv a license to display the preprint in perpetuity. It is made available under aCC-BY 4.0 International license.

1088 *Performance of parameter estimation using Submodel 5*

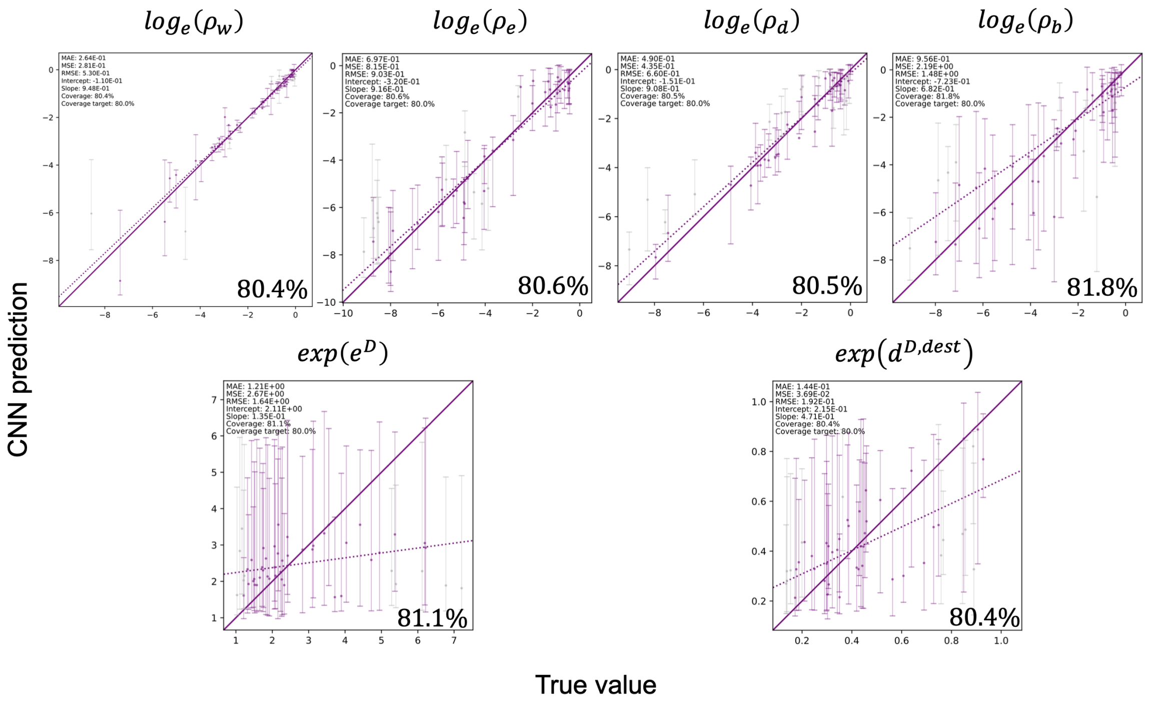

Figure 16: Plots showing 80% CPIs for each parameter on the test dataset simulated under submodel 5. The $x-$axis is showing the true parameter values, and the $y-$axis is showing the estimated values from CNN. All test data were used for the regression. Of these, point estimates (markers) and 80% CPIs (bars) are shown for 50 examples.

bioRxiv preprint doi: https://doi.org/10.64898/2026.02.17.706216; this version posted February 18, 2026. The copyright holder for this preprint (which was not certified by peer review) is the author/funder, who has granted bioRxiv a license to display the preprint in perpetuity. It is made available under aCC-BY 4.0 International license.

Performance of parameter estimation using Submodel 6

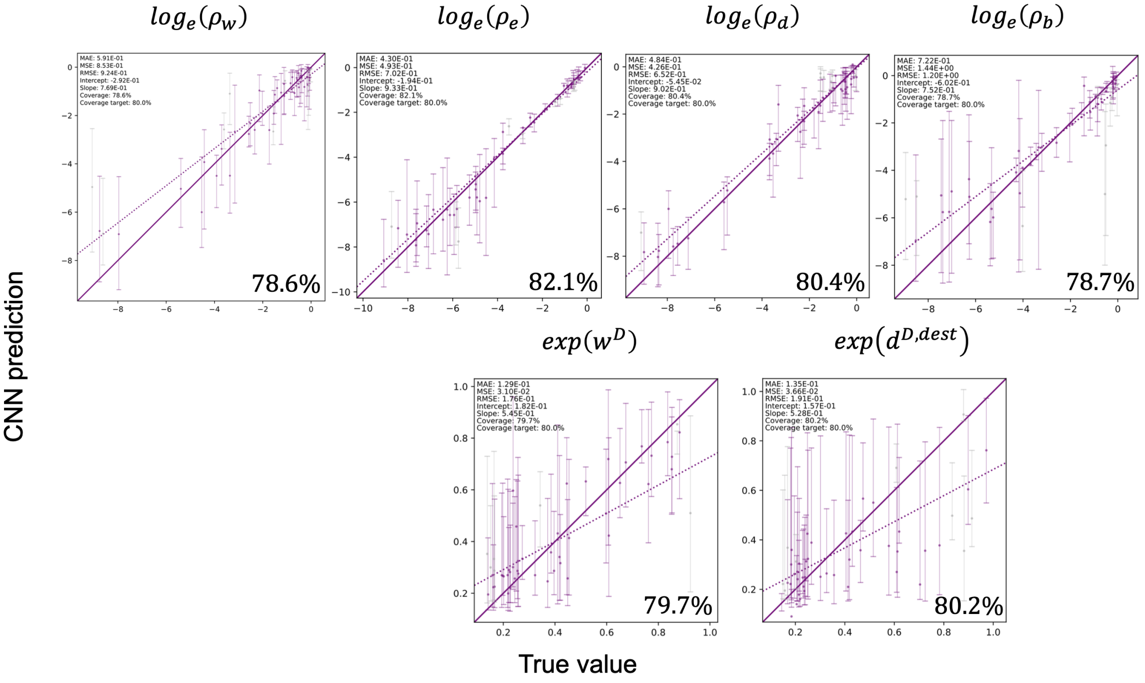

Figure 17: Plots showing 80% CPIs for each parameter on the test dataset simulated under submodel 6. The $x-$axis is showing the true parameter values, and the $y-$axis is showing the estimated values from CNN. All test data were used for the regression. Of these, point estimates (markers) and 80% CPIs (bars) are shown for 50 examples.

bioRxiv preprint doi: https://doi.org/10.64898/2026.02.17.706216; this version posted February 18, 2026. The copyright holder for this preprint (which was not certified by peer review) is the author/funder, who has granted bioRxiv a license to display the preprint in perpetuity. It is made available under aCC-BY 4.0 International license.

Deep learning accuracy for empirical estimation

	Estimate	*Anolis*		*Viburnum*	
Parameter estimation for best-fitting model	ρ_w	0.2724	$[0.1049, 0.6722]$	1.6401	$[0.5831, 2.4766]$
	ρ_e	0.0003	$[0.0001, 0.0016]$	0.0021	$[0.00063, 0.0161]$
	ρ_d	0.0009	$[0.0002, 0.0029]$	0.0021	$[0.00056, 0.0686]$
	ρ_b	0.0842	$[0.0005, 0.3391]$	0.0266	$[0.00063, 2.8511]$
	w^D	-1.55	$[-1.83, -0.79]$	-1.90	$[-2.18, -0.77]$
	e^D	0.80	$[0.21, 1.76]$	–	–
	$d^{D,dest}$	-1.25	$[-1.87, -0.36]$	-1.30	$[-2.00, -0.37]$
**Validation loss score**		0.609		0.487	

Table 6: DDGeoSSE estimates for Caribbean *Anolis* lizards and Neotropical *Oreinotinus* plants using phyddle. Rows 1-7 show point estimates and 80% CPIs (in brackets) for the best-fitting model. Rows 1-4 show base rates for within-region speciation (ρ_w), extinction (ρ_e), dispersal (ρ_d), and between-region speciation (ρ_b). Rows 5-7 show diversity-dependent effect parameters for within-region speciation $\left(w^D\right)$, extinction $\left(e^D\right)$, and incoming dispersal $\left(d^{D,dest}\right)$. Note that the base rates for *Viburnum* have been converted to correspond to the original phylogeny (branch lengths are measured in unit time). The last column shows the loss score calculated from each model's corresponding validation dataset. The empirical estimates and loss score from each model are comparable to the results shown by another set of independent networks trained on the same datasets on Table 4.

bioRxiv preprint doi: https://doi.org/10.64898/2026.02.17.706216; this version posted February 18, 2026. The copyright holder for this preprint (which was not certified by peer review) is the author/funder, who has granted bioRxiv a license to display the preprint in perpetuity. It is made available under aCC-BY 4.0 International license.

Deep learning accuracy for model selection using simulated dataset – I

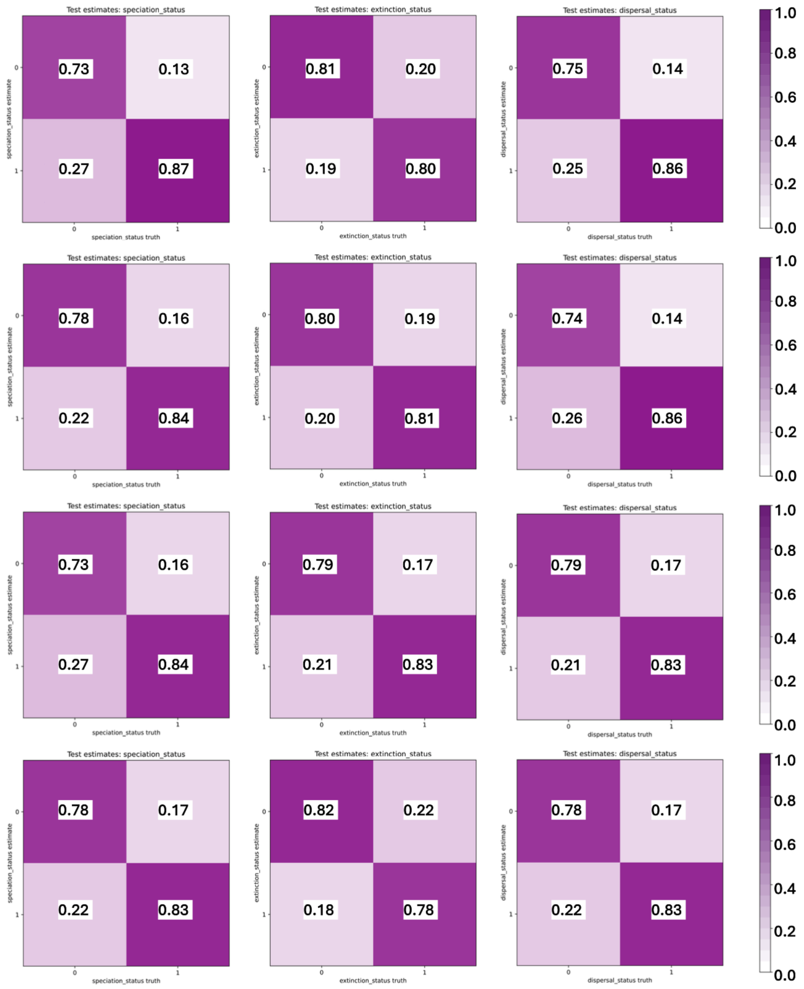

Figure 18: Performance on the test dataset using four independently trained networks for each training target on 400,000 simulations, generated by 8 submodels as described in Table 2 (50,000 each), for detecting the presence (1) or absence (0) of diversity dependence in within-region speciation (left panel), extinction (middle panel), and dispersal (right panel) separately. In total, we have 24 independently trained networks. The $x-$axis shows the true scenario, and the $y-$axis shows the predicted scenario.

bioRxiv preprint doi: https://doi.org/10.64898/2026.02.17.706216; this version posted February 18, 2026. The copyright holder for this preprint (which was not certified by peer review) is the author/funder, who has granted bioRxiv a license to display the preprint in perpetuity. It is made available under aCC-BY 4.0 International license.

Deep learning accuracy for model selection using simulated dataset – II

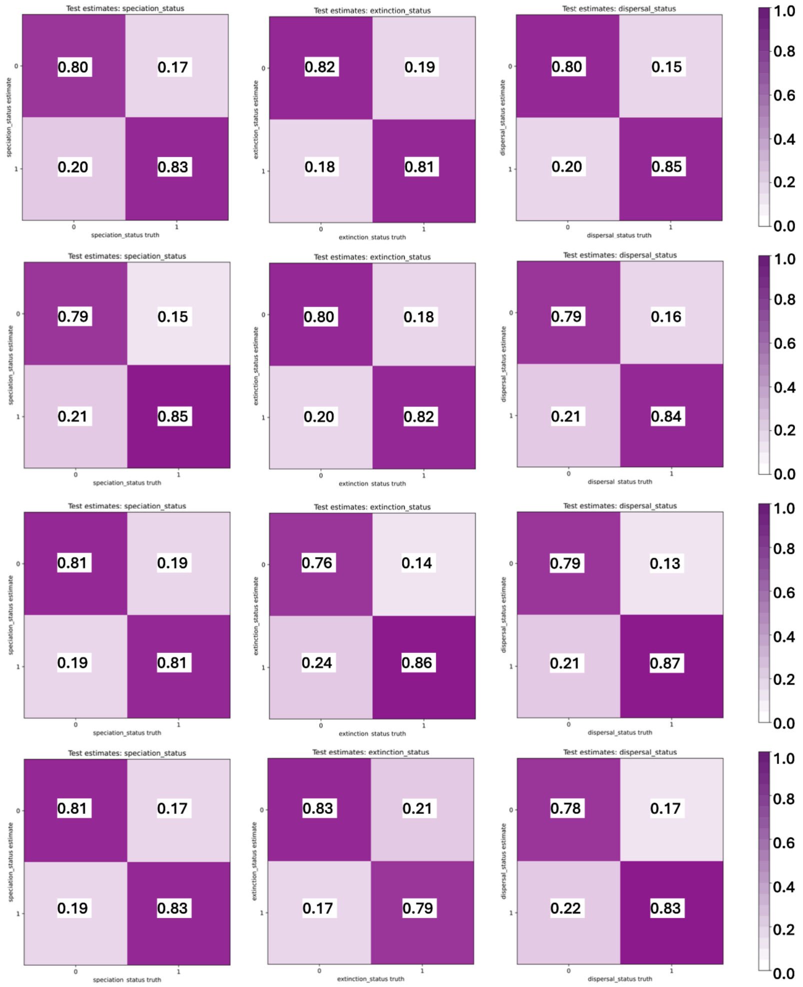

Figure 19: Performance on the test dataset using another set of four independently trained networks for each training target on 400,000 simulations, generated by 8 sub-models as described in Table 2 (50,000 each), for detecting the presence (1) or absence (0) of diversity dependence in within-region speciation (left panel), extinction (middle panel), and dispersal (right panel) separately. In total, we have 24 independently trained networks. The $x-$axis shows the true scenario, and the $y-$axis shows the predicted scenario.

bioRxiv preprint doi: https://doi.org/10.64898/2026.02.17.706216; this version posted February 18, 2026. The copyright holder for this preprint (which was not certified by peer review) is the author/funder, who has granted bioRxiv a license to display the preprint in perpetuity. It is made available under aCC-BY 4.0 International license.

1093 *Phylogenies and biogeography of empirical systems*

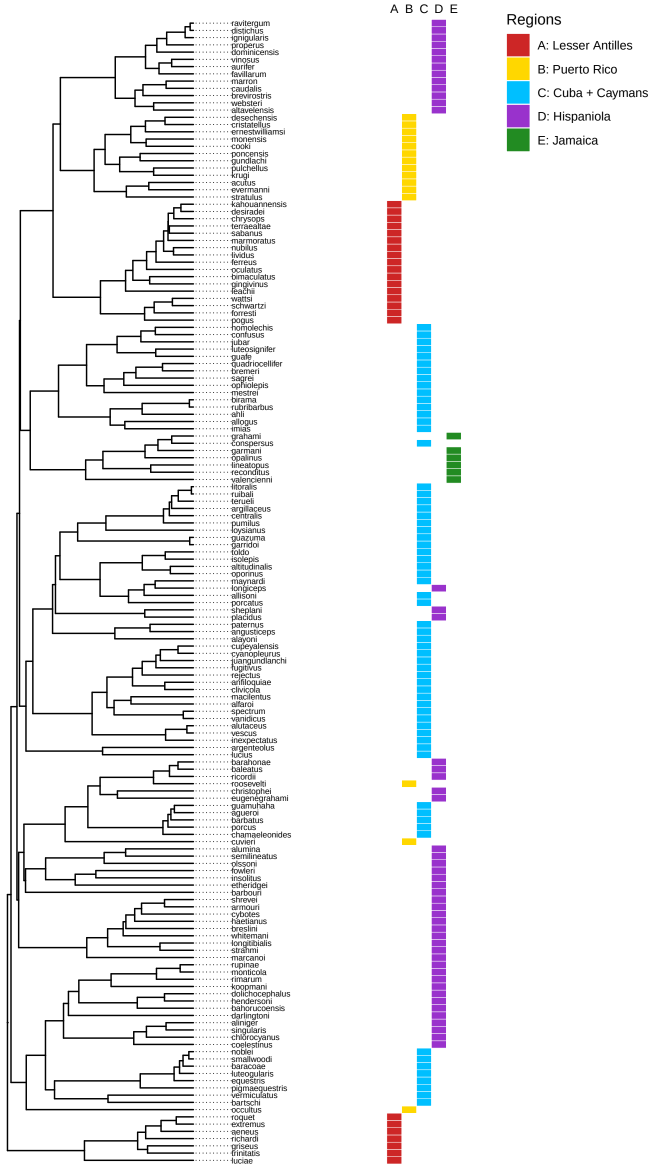

Figure 20: Phylogeny and species ranges for 158 *Anolis* lizard species inhabiting Caribbean islands. The phylogeny was subsampled from the tree produced by Poe et al. (2017). Ranges from Poe et al. (2017) were recoded for 5 island regions.

bioRxiv preprint doi: https://doi.org/10.64898/2026.02.17.706216; this version posted February 18, 2026. The copyright holder for this preprint (which was not certified by peer review) is the author/funder, who has granted bioRxiv a license to display the preprint in perpetuity. It is made available under aCC-BY 4.0 International license.

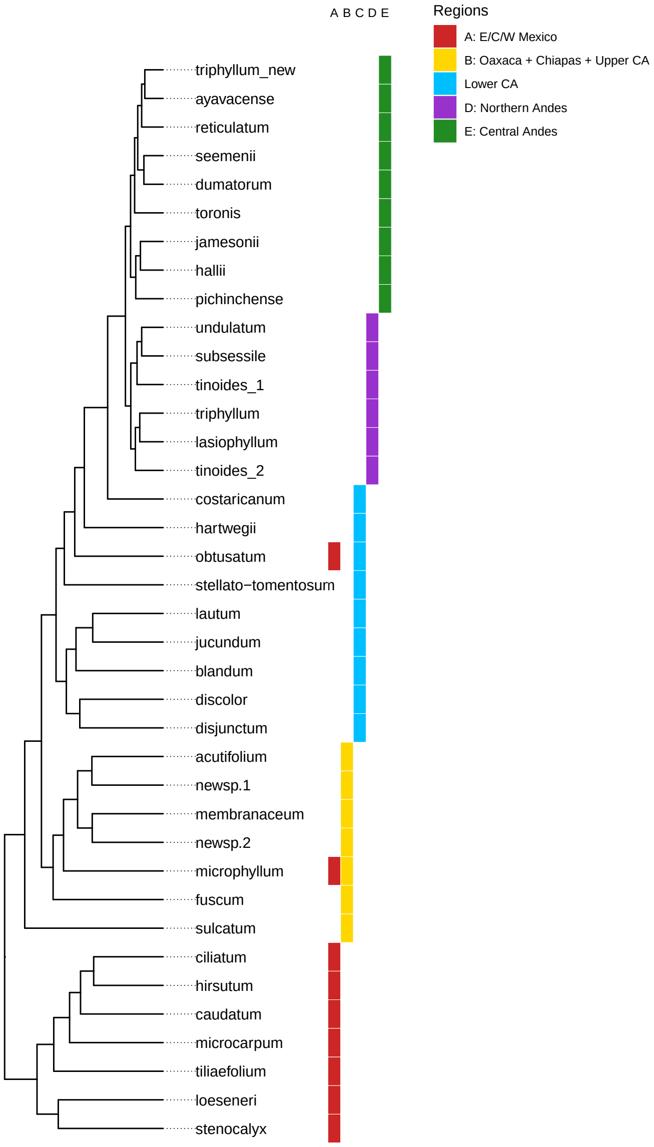

Figure 21: Phylogeny and species ranges for 38 *Oreinotinus* (a clade within *Viburnum*) plant species inhabiting neotropical cloud forests. The phylogeny was subsampled from the tree produced by Donoghue et al. (2022). Ranges from Donoghue et al. (2022) were recoded for 5 montane regions.

bioRxiv preprint doi: https://doi.org/10.64898/2026.02.17.706216; this version posted February 18, 2026. The copyright holder for this preprint (which was not certified by peer review) is the author/funder, who has granted bioRxiv a license to display the preprint in perpetuity. It is made available under aCC-BY 4.0 International license.

Quality of trained networks for empirical parameter estimation

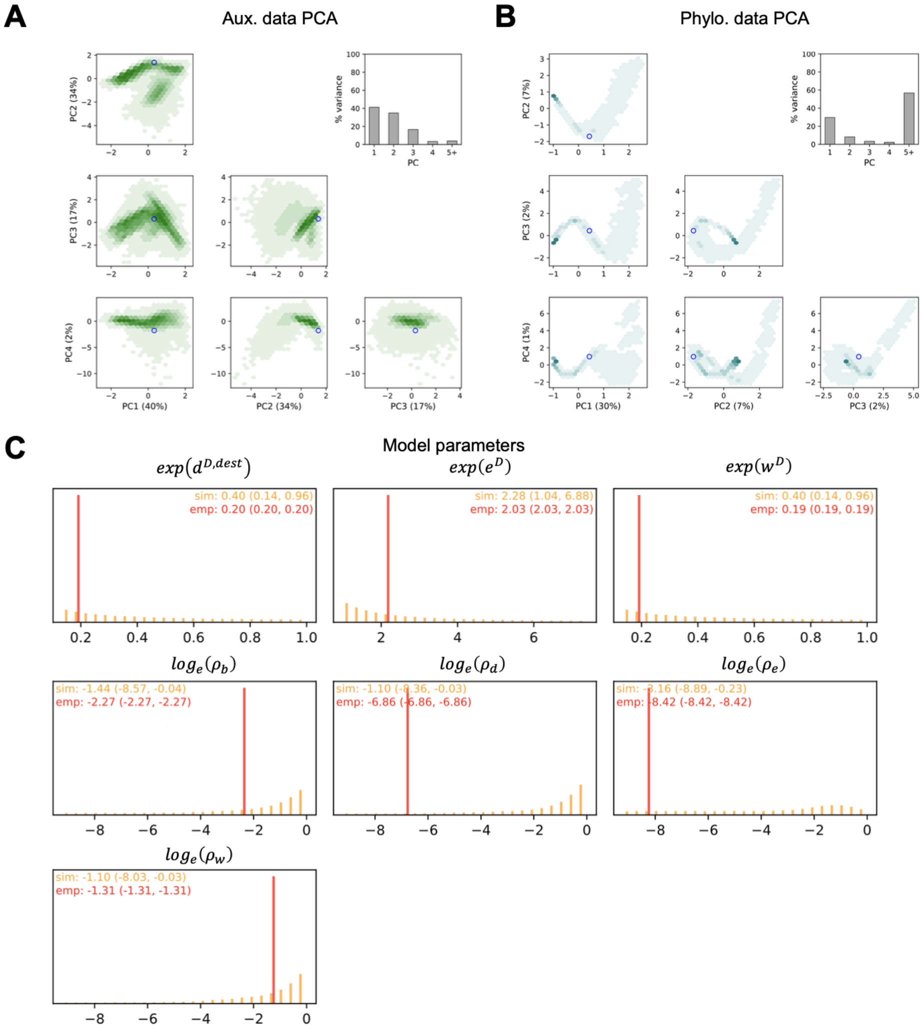

Figure 22: Network for *Anolis* analysis. PCA plots for the auxiliary data tensor (a) and phylogenetic data tensor (b) training sets. The empty circle represents the *Anolis* dataset in the PCA space. Histogram of Log-DDG model parameters in the training set (c). The red line represents the *Anolis* parameter estimates.

bioRxiv preprint doi: https://doi.org/10.64898/2026.02.17.706216; this version posted February 18, 2026. The copyright holder for this preprint (which was not certified by peer review) is the author/funder, who has granted bioRxiv a license to display the preprint in perpetuity. It is made available under aCC-BY 4.0 International license.

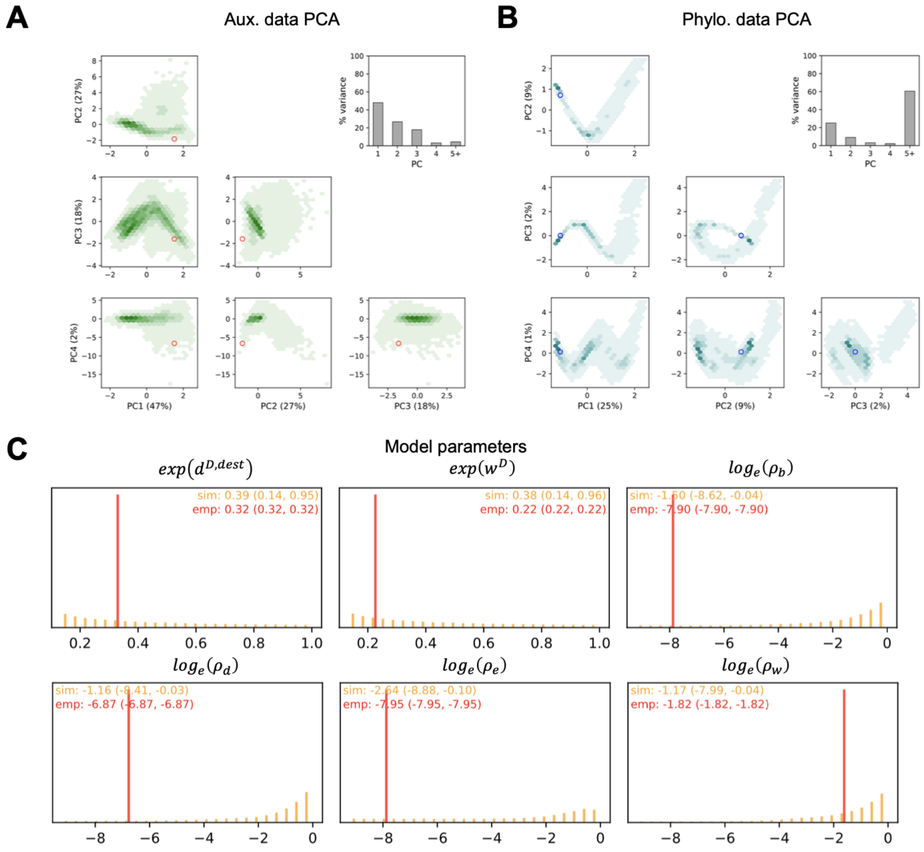

Figure 23: Network for *Viburnum* analysis. PCA plots for the auxiliary data tensor (a) and phylogenetic data tensor (b) training sets. The empty circle represents the *Viburnum* dataset in the PCA space. Histogram of Log-DDG model parameters in the training set (c). The red line represents the *Viburnum* parameter estimates.

bioRxiv preprint doi: https://doi.org/10.64898/2026.02.17.706216; this version posted February 18, 2026. The copyright holder for this preprint (which was not certified by peer review) is the author/funder, who has granted bioRxiv a license to display the preprint in perpetuity. It is made available under aCC-BY 4.0 International license.

Julia simulator validation using GeoSSE model

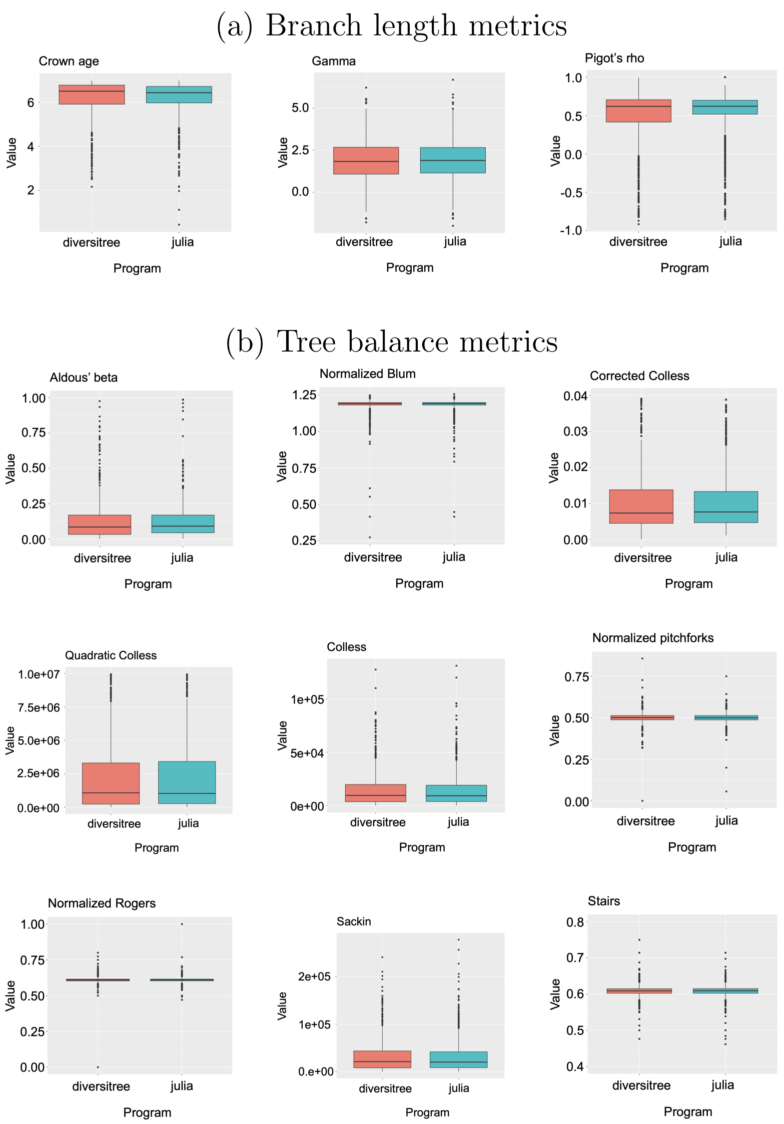

Figure 24: Comparison of tree balance and branch length metrics between our Julia script and diversitree for simulating GeoSSE trees without model misspecification. For each program, we simulated 1,000 trees using the following parameters: $w_A = 1$, $w_B = 0.5$, $b_B^A = 0.5$, $e_A = 0.2$, $e_B = 0.4$, $d_{AB} = 2.5$, $d_{BA} = 0.5$. Each row shows related metrics as indicated by the bold row headings.

bioRxiv preprint doi: https://doi.org/10.64898/2026.02.17.706216; this version posted February 18, 2026. The copyright holder for this preprint (which was not certified by peer review) is the author/funder, who has granted bioRxiv a license to display the preprint in perpetuity. It is made available under aCC-BY 4.0 International license.

(a) Branch length metrics

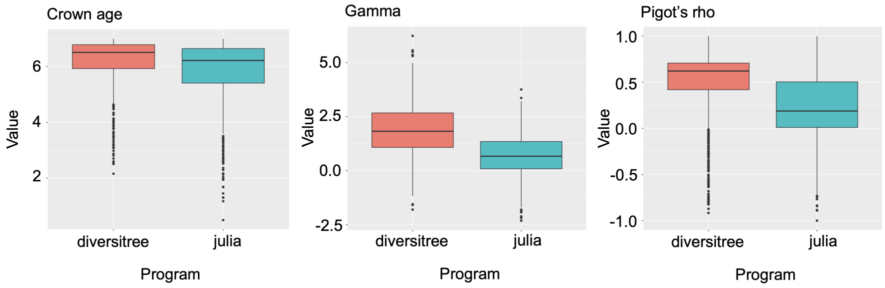

(b) Tree balance metrics

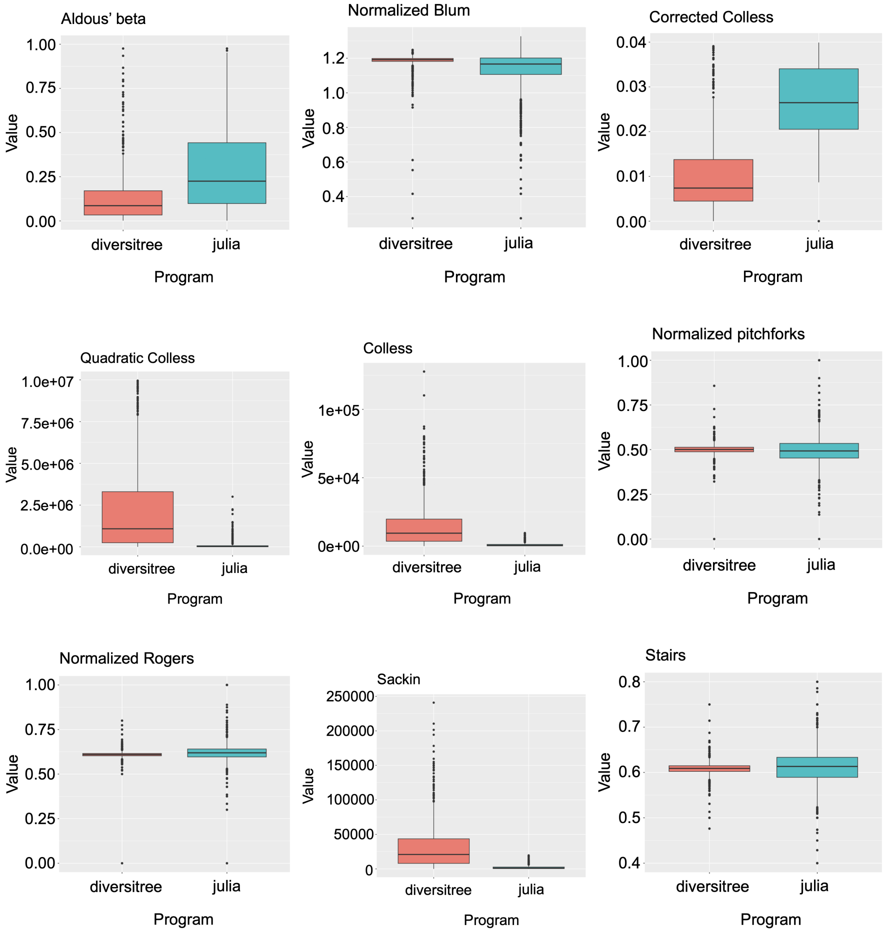

Figure 25: Comparison of tree balance and branch length metrics between our Julia script and diversitree for simulating GeoSSE trees with intentional model misspecification, where the speciation rates in both regions are doubled for Julia simulations to cause the results to differ. We simulated 1000 trees using the following parameters: $w_A = 1,\ w_B = 0.5,\ b_B^A = 0.5,\ e_A = 0.2,\ e_B = 0.4,\ d_{AB} = 2.5,\ d_{BA} = 0.5.$

bioRxiv preprint doi: https://doi.org/10.64898/2026.02.17.706216; this version posted February 18, 2026. The copyright holder for this preprint (which was not certified by peer review) is the author/funder, who has granted bioRxiv a license to display the preprint in perpetuity. It is made available under aCC-BY 4.0 International license.

Neural network architecture

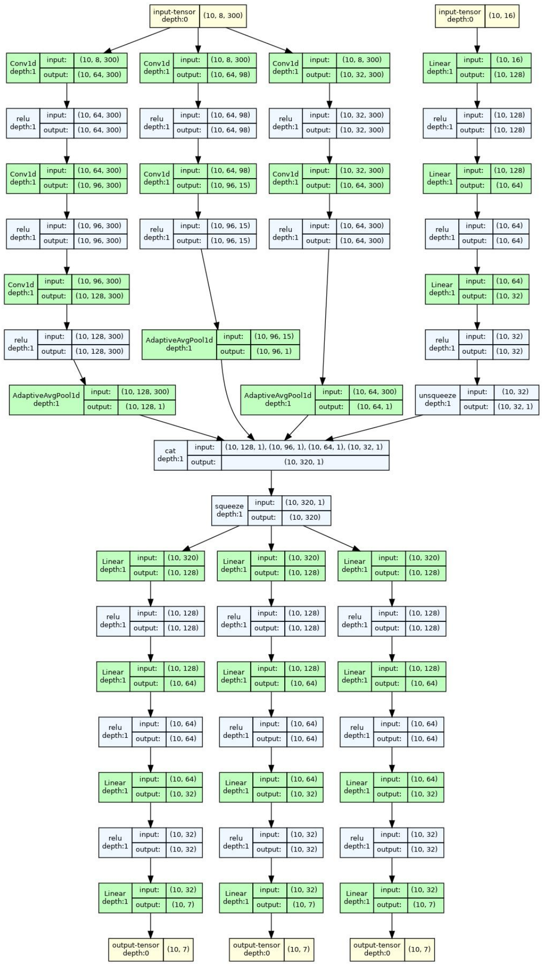

Figure 26: Neural network architecture used for parameter estimation tasks with `phyddle`. This architecture corresponds to Log-DDG submodel 7, with seven outputs corresponding to the four base rate parameters (ρ_e, ρ_w, ρ_d, ρ_b) plus three diversity-dependent effect parameters (e^D, w^D, $d^{D,dest}$). All other submodels used the same architecture, except with fewer outputs for those effect parameters that were fixed to 0 (no effect). Green boxes correspond to nodes, blue boxes correspond to activation functions, yellow boxes correspond to inputs and outputs. Arrows show how information is passed between layers, with numbers in parentheses describing tensor shapes.

bioRxiv preprint doi: https://doi.org/10.64898/2026.02.17.706216; this version posted February 18, 2026. The copyright holder for this preprint (which was not certified by peer review) is the author/funder, who has granted bioRxiv a license to display the preprint in perpetuity. It is made available under aCC-BY 4.0 International license.

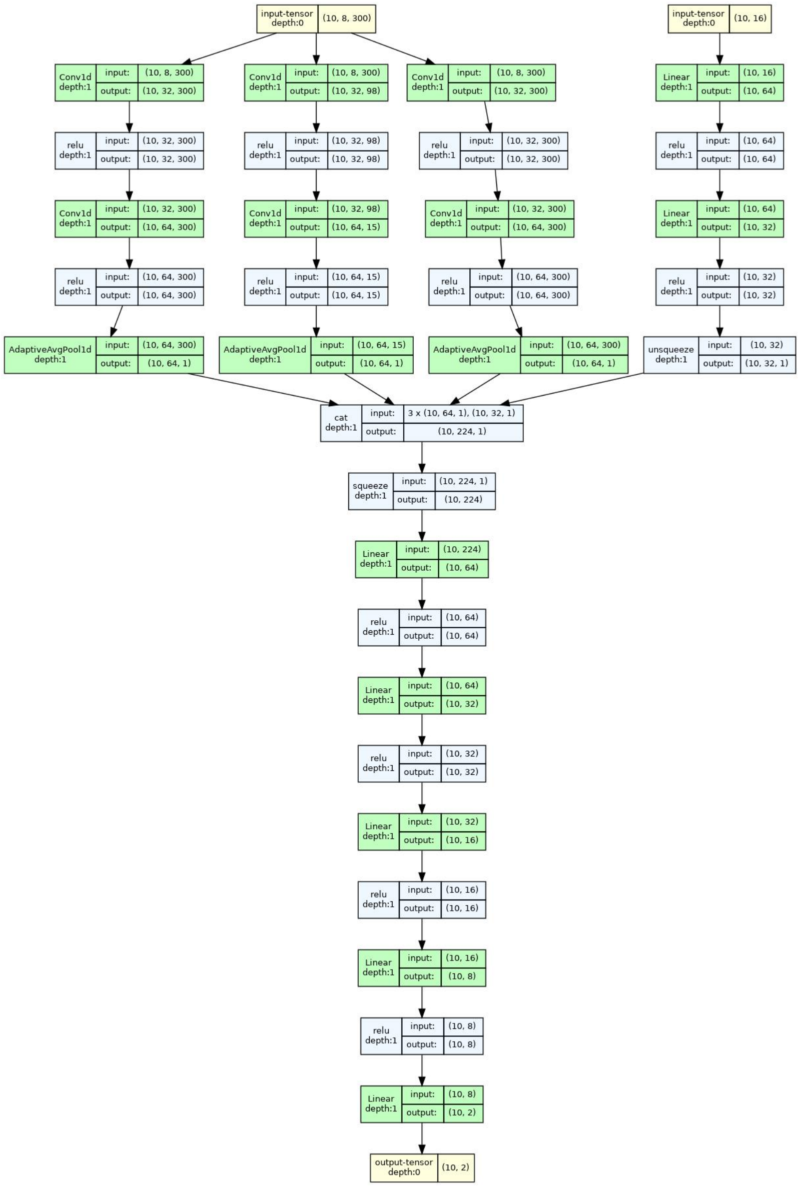

Figure 27: Neural network architecture used for model selection tasks with `phyddle`. While this particular example was used for model selection for diversity-dependent dispersal (to test whether $d^{D,dest} = 0$ or $d^{D,dest} \neq 0$), the exact same architecture was used in separately trained networks for model selection for diversity-dependent within-region speciation (w^D) and extinction (e^D). Green boxes correspond to nodes, blue boxes correspond to activation functions, yellow boxes correspond to inputs and outputs. Arrows show how information is passed between layers, with numbers in parentheses describing tensor shapes.
